# Natural Regeneration in the Tumbesian Dry Forest: Identification of the Drivers Affecting Abundance and Diversity

**DOI:** 10.1038/s41598-020-66743-x

**Published:** 2020-06-17

**Authors:** Jorge Cueva-Ortiz, Carlos Iván Espinosa, Zhofre Aguirre-Mendoza, Elizabeth Gusmán-Montalván, Michael Weber, Patrick Hildebrandt

**Affiliations:** 1 Institute of Silviculture, TUM School of Life Sciences Weihenstephan, Technical University of Munich, Freising, 85354 Germany; 2 EcoSs_Lab, Departamento de Ciencias Biológicas, Universidad Técnica Particular de Loja, San Cayetano Alto, Loja, 110107 Ecuador; 3 Carrera de Ingeniería Forestal, Universidad Nacional de Loja, Ciudadela Guillermo Falconí E., Loja, 110-110 Ecuador

**Keywords:** Biodiversity, Forestry, Forest ecology

## Abstract

Tropical and subtropical dry forests make up the world’s largest terrestrial ecosystem. However, these forests have been used to establish several productive activities, such as growing crops, rearing livestock, and using the forest resources, due to their ease of access and climatic conditions, which has led to this ecosystem becoming highly threatened. Therefore, this research assessed the effects of anthropogenic pressures and a number of abiotic variables on natural regeneration in dry forests in the Tumbesian region by addressing three research questions: (a) What is the status of natural regeneration in terms of abundance and diversity? (b) Does livestock grazing and the anthropogenic pressure affect the abundance and diversity of natural regeneration? (c) Does seasonality or grazing have the greatest influence on the regeneration dynamics? Data were obtained from 72 samples (36 fenced and 36 unfenced) during five surveys spanning a 2-year period, and the seedling abundance, mortality, recruitment, species richness and diversity were evaluated using linear mixed models. Natural regeneration was most positively affected by rainy season precipitation, but soil conditions also played an important role. Short-term fences had a major effect on reducing mortality but did not improve the abundance or diversity, whereas cattle grazing significantly affected the abundance of seedlings.

## Introduction

Scientists have been depicting the threats that tropical dry forests are exposed to for many years^1–6^ and have identified some parts of this ecosystem as places of tremendous diversity and endemism^7–9^. These insights have supported the establishment of new protected areas in several regions of the world–for instance, the Brazilian Minister of the Environment identified 52 priority areas for conservation in the Caatinga in 2002, 27 of which were catalogued as an extreme priority^10^; Koleff *et al*.^11^ stated in 2012 that 42% of the Mexican tropical dry forests should be protected; several private and state-owned areas were added to the list of protected areas in Ecuador, including the ‘Bosques de Paz’ Biosphere Reserve, in 2017^12^; and one of the most representative areas of Peru was placed under protection by the Amazonas Department in 2018^13^. However, although the number of protected areas has increased and a large amount of research has been undertaken on the biophysical aspects of tropical dry forests of Latin America, Caribbean and Africa^14,15^, little is known about the ecology and regeneration of tropical dry forests^16^ or the effects of domestic animals and other threats on the natural regeneration of these forests, which are still diminishing in size^14^.

The tropical dry forest that extends along the Pacific coast from the southwest of Ecuador to the northwest of Peru covers approximately 64,500 km^2^ ^17^ and faces similar issues^18^. This region, which is named the ‘Tumbesian region’, is known for its high level of endemism among woody species^7,19^ and for being one of the better preserved areas in the region^20,21^. Some effort has been made to understand the ecosystem functionality and the effects of animals on the forest in this region, examples of which include the studies of Jara-Guerrero *et al*.^22^, Espinosa *et al*.^18,23,24^, Piana & Marsden^25^ and Cueva *et al*.^26^, who evaluated the influence of environmental conditions, anthropogenic disturbances or soil characteristics on seed dispersal or composition of mature dry forest. However, little attention has been paid to the natural regeneration, which has rarely been studied in the region (neither that generated by sprouts nor by seeds), despite this being a key component of the sustainability of the forest^27^. Studies that have been conducted include an assessment of the effect of goats in one village of Ecuador by Rodriguez in 2006^28^, a very short report on a protected area in Peru by Abou *et al*. in 2010^29^, and a structural and compositional characterisation of regeneration in three dry forest types in Ecuador by Aguirre *et al*. in 2013^30^.

As with most tropical dry forests, natural regeneration in the Tumbesian region is affected by seasonality, which is characterised by a long period of drought for 7 months of the year^31^, as well as by human activities, particularly the grazing of domestic animals^20^. However, the extent to which natural regeneration is affected by these biotic and abiotic factors remains poorly understood. Therefore, in this study, we attempted to fill these knowledge gaps by addressing the following research questions: (a) What is the status of natural regeneration in the dry forest in terms of abundance and diversity, taking into account seasonality and grazing? (b) Do livestock grazing and other anthropogenic pressures affect the abundance and diversity of natural regeneration in the dry forest? (c) Does seasonality or grazing have the greatest influence on the dynamics of natural regeneration in the dry forest?

## Results

### Status of natural regeneration

**Figure 1:**
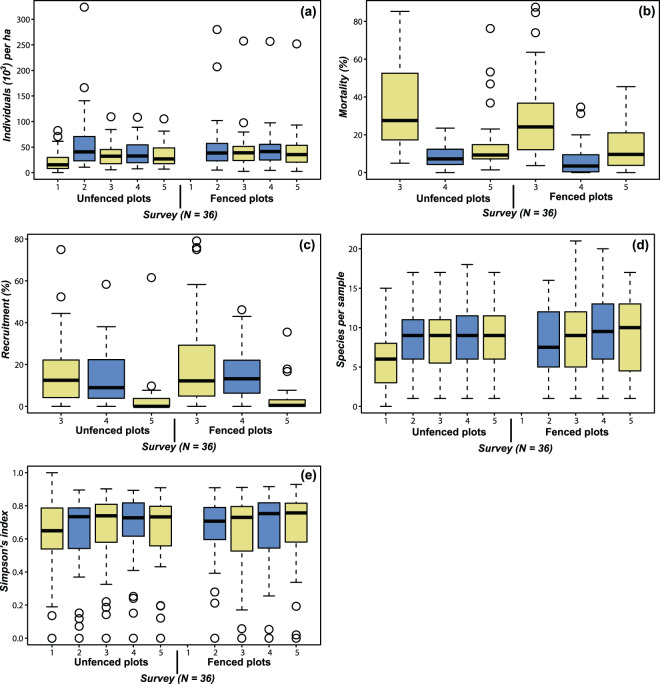
Changes in the natural regeneration parameters through time and under different treatments. **(a)** Abundance, **(b)** mortality, **(c)** recruitment, **(d)** species richness and **(e)** diversity of seedlings on each survey date in unfenced and fenced plots. Fenced plots were not assessed in survey 1 because they were selected based on the first evaluation of the unfenced plots. Numbers from 1 to 5 correspond to consecutive surveys, which spanned a 2-year period. Yellow = dry season, blue = rainy season.

Measurements were made across a total of 1,152 m^2^ of dry forest during five surveys (four in fenced plots), that were carried out over a nearly 2-year period. The total number of seedlings recorded in fenced plus unfenced plots in the evaluated area were: 6,280; 4,751; 5,069 and 4,572 individuals in surveys 2, 3, 4 and 5 respectively. The average number of seedlings per plot projected to one hectare and computed for each survey varied from 21,900 to 57,200 individuals in unfenced plots and from 43,500 to 51,800 individuals per ha in fenced plots (Fig. 1a). The average abundance decreased slightly from the rainy season to the dry seasons, but remained relatively constant when measurements from the same season and treatment were compared. This seasonal effect was less pronounced in the fenced plots than the unfenced plots due to the lower dry season mortality (Fig. 1b) resulting in similar numbers of individuals occurring in the two seasons (median number of individuals per ha = 35,300–41,200 in fenced plots and 26,900–40,600 in unfenced plots).

In the first rainy season, *Simira ecuadorensis* (Standl.) Steyerm and *Erythroxylum glaucum* O. E. Schulz had the highest abundances in the study area (1,447 and 1,371 individuals, respectively). *Simira ecuadorensis* showed good resistance to the dry season, with 1,561 individuals being recorded in the final survey. By contrast, most *E. glaucum* individuals died during the dry season, with only 265 individuals being recorded alive in the final survey.

During the monitoring period, the mortality of plants was influenced by seasonality, increasing from an average of 9.0% in the rainy season to 35.8% in the dry season, with the same pattern being observed across both treatments (Fig. 1b). However, mortality was slightly lower in the fenced plots than in the unfenced plots. Substantial recruitment was recorded in the fenced plots in surveys 3 and 4 (21.4% and 16.6%, respectively), whereas recruitment was very low in the final survey (3.1%). By contrast, recruitment decreased over time from 16.1% to 3.7% in the unfenced plots, even during the rainy season (Fig. 1c).

A total of 85 species from 31 families were recorded in the study area (see Supplementary Table S1), which included 37 tree species, 36 shrub species and 12 species that could not be identified. The number of species remained almost the same across surveys 2 to 5 in the unfenced plots (mean = 8.8, 8.6, 9.1 and 8.7 species per plot, respectively) but showed a slight increase over time in the fenced plots (mean = 8.4, 8.8, 9.6 and 9.4 species per plot) (Fig. 1d). A similar pattern was also observed for Simpson’s index, which had mean values ranging from 0.62 in survey 1 to 0.67 in survey 4 for the unfenced plots and 0.62 in survey 3 to 0.66 in survey 4 for the fenced plots (Fig. 1e). Surprisingly, during the evaluation period no seedlings were recorded for some of the characteristic species^32^ of the study area, including *Ceiba trischistandra* (A.Gray) Bakh., *Cavanillesia platanifolia* (Bonpl.) Kunth, *Eriotheca roseorum* (Cuatrec.) A.Robyns and *Myroxylon balsamum* (L.) Harms. Furthermore, only a few individuals of other species were recorded, such as *Centrolobium ochroxylum* Rose ex Rudd (one individual) and *Loxopterygium huasango* Spruce ex Engl. (two individuals).

### Effects of biotic and abiotic factors on natural regeneration

Model selection revealed that the amount of variance in abundance that could be explained by the best models ranged from 10% to 13% for the fixed effects and from 56% to 60% for the fixed + random effects. Abundance appears to be more influenced by pressure predictors (Table 1). By contrast, the amount of variance in species richness that could be explained by the best models ranged from 51% to 63% for the fixed effects and equated to 72% for the fixed + random effects. The diversity was explained only by the soil predictors; just one model was selected, which explained 40% of the variance using the fixed effects and 53% of the variance using both fixed + random effects.

**Table 1:** Best models explaining the influence of biotic and abiotic factors on the response variables abundance, species richness and diversity.

Model	df	AIC	∆AIC	*R2m*	*R2c*
**Abundance**					
~1 + Equine * Treat + SPrec + Cattle	8	3158.11	0.00	0.10	0.57
~1 + Goats * Cattle * Equine * Treat + SPrec	19	3159.10	0.99	0.13	0.60
~1 + Equine * Treat + Cattle * Treat + SPrec	9	3159.94	1.83	0.10	0.57
~1 + Equine * Treat + SPrec	7	3159.97	1.86	0.10	0.56
**Species richness**					
~1 + SPrec + Treat + SDepth + Drain	12	1530.18	0.00	0.63	0.72
~1 + SPrec + SDepth + Drain	11	1530.45	0.28	0.62	0.72
~1 + Equine + SPrec + Treat + SDepth + Drain	13	1531.73	1.56	0.63	0.72
~1 + Cattle + SPrec + Treat + Text	10	1531.82	1.65	0.51	0.72
~1 + Equine + SPrec + SDepth + Drain	12	1532.01	1.83	0.63	0.72
**Diversity**					
~1 + Drain + Text	8	−233.70	0.00	0.40	0.53

Linear mixed models were computed using a negative binomial error distribution for abundance, the Poisson distribution with the Laplace approximation for species richness and the restricted maximum likelihood (REML) approach for diversity. Models were selected and ordered according to the delta Akaike information criterion (∆AIC < 2). *R2m* and *R2c* represent the amount of variance that was explained by the fixed effects and fixed + random effects, respectively.

Four models were identified as best explaining the abundance of natural regeneration (Table 1). All of these models included rainy season precipitation (SPrec) and three included the interaction between the presence of horses and donkeys (Equine) and treatment (Treat). Only the best model included the presence of cows and bulls (Cattle) as an independent predictor, while the second and third best model include this predictor as a term of interaction. The four-way interaction between the presence of goats (Goats), Cattle, Equine and Treat was included in the second-best model, and the interaction between Cattle and Treat was included in the third-best model.

**Figure 2:**
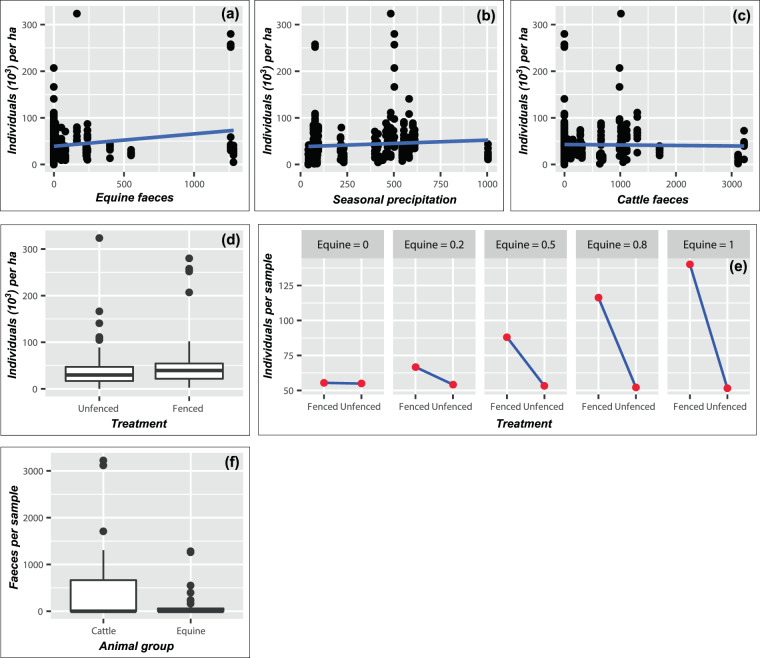
Relationship between the abundance of regenerating seedlings and the predictors. **(a)** equines, **(b)** seasonal precipitation, **(c)** cattle, **(d)** treatment and **(e)** the treatment–equine interaction. **(f)** A comparison of the amounts of cattle and equine faeces in the study area, presented as dry weights.

Both Equine and SPrec had significant positive effects on the abundance of natural regeneration, with the latter having the most evident positive effect (Table 2; Fig. 2a,b). By contrast, Cattle had a significant negative effect on abundance (Table 2; Fig. 2c), indicating that natural regeneration is affected by the grazing of cows and bulls. While fences (Treatopen) did not affect the number of individuals when it was used as main predictor (Fig. 2d), the interaction Treat and Equine had a significant negative effect (Table 2; Fig. 2e), demonstrating that the abundance of regenerating seedlings was lower in unfenced sites where horses were present than in fenced sites where horses had previously been present.

**Table 2:** Effects of structure and diversity predictors on the response variables abundance, species richness and diversity in the best models.

Predictor	Estimate	Std. Error	*p* (<0.05)
**Abundance**			
(Intercept)	3.17	0.25	<2.0 × 10^−16^***
Equine	0.93	0.23	7.0 × 10^−5^***
Treatopen	−0.01	0.07	0.89
SPrec	0.39	0.08	5.7 × 10^−7^***
Cattle	−0.37	0.18	0.05*
Equine:Treatopen	−0.99	0.27	3.1 × 10^−4^***
**Species richness**			
(Intercept)	−0.15	0.30	0.61
SPrec	0.15	0.05	3.6 × 10^−3^**
Treatopen	−0.06	0.04	0.13
SDepth11–20 cm	1.32	0.28	2.2 × 10^−6^***
SDepth21–50 cm	1.93	0.27	1.2 × 10^−12^***
SDepth51–100 cm	1.45	0.26	1.7 × 10^−8^***
SDepth >100 cm	2.06	0.29	6.1 × 10^−13^***
DrainModerated	0.45	0.16	0.01**
DrainGood	0.28	0.14	0.05*
**Diversity**			
(Intercept)	0.19	0.07	0.01**
DrainModerated	0.16	0.08	0.04*
DrainGood	−0.05	0.08	0.52
TextClay loam	0.17	0.08	0.04*
TextLoam	0.22	0.06	2.0 × 10^−4^***
TextSandy loam	−0.12	0.10	0.27

The *p-*values of 0.05 for Cattle as a predictor of abundance and DrainGood as a predictor of species richness were due to rounding. ***p < 0.001; **p < 0.01; *p < 0.05.

Species richness was best explained by five models, all of which included SPrec, four of which included soil depth (SDepth) and drainage (Drain), and three of which included Treat (Table 1). All of these predictors were included in the best model.

SPrec had a significant positive influence on species richness (Table 2), indicating that species richness was higher in the rainy season. Species richness was also significantly higher in sites with deeper soils than in sites with shallow soils (<10 cm) and in sites with moderately and well-drained soils than in sites with compacted or non-permeable soils (Table 2). As seen for abundance, fences did not affect the species richness in our study area.

Only one model was selected for diversity, which included soil drainage and texture (Text) as predictors (Table 1). Moderately drained soils had a significant positive effect on the diversity compared with poorly drained soils, and sites with clay loam and loam soils had a significantly higher diversity than those with clay–sandy loam soils (Table 2).

Regarding the dynamics of natural regeneration, 36% and 56% of the variance in mortality was explained by the fixed and fixed + random effects, respectively, that were included in the best model, while 30–35% and 60–61% of the variance in recruitment was explained by the fixed and fixed + random effects, respectively (Table 3).

**Table 3:** Best models explaining the dynamics of natural regeneration. Linear mixed models were computed using the restricted maximum likelihood (REML) approach with a Bayesian fit for mortality and a Gaussian error distribution for recruitment.

Model	df	AIC	∆AIC	*R2m*	*R2c*
**Mortality**					
~1 + SPrec + Treat + Time	8	587.18	0.00	0.36	0.56
**Recruitment**					
~1 + SPrec * Time + Treat	10	657.59	0.00	0.35	0.61
~1 + SPrec + Time	8	659.20	1.62	0.30	0.60

Models were selected and ordered according to the delta Akaike information criterion (∆AIC < 2). *R2m* and *R2c* represent the amount of variance that was explained by the fixed effects and fixed + random effects, respectively.

Only one model was identified as best explaining mortality, which included SPrec, Treat and elapsed time (Time) as fixed effects (Table 3). Both SPrec and Time had significant negative effect on mortality (Table 4), indicating that mortality was lower in the rainy season than in the dry season and also decreased with elapsed time through the monitoring period. In addition, the mortality of natural regeneration was significantly higher in unfenced plots than in the fenced plots.

**Table 4:** Effects of predictors of mortality and recruitment that were included in the best models.

Predictor	Estimates	Std. Error	*p* (<0.05)
**Mortality**			
(Intercept)	8.02	0.59	
SPrec	−1.61	0.15	<2.2 × 10^−16^***
Treatopen	0.33	0.11	3.7 × 10^−3^**
Time	−0.11	0.02	3.0 × 10^−5^***
**Recruitment**			
(Intercept)	−9.35	5.52	0.09^†^
SPrec	7.67	2.80	0.01**
Time	0.63	0.32	0.05^†^
Treatopen	−0.10	0.14	0.44
SPrec:Time	−0.42	0.16	0.01*

***p < 0.001; **p < 0.01; *p < 0.05; ^†^*p* < 0.1.

The two best models explaining recruitment both included SPrec and Time as predictors (Table 3). While both of these predictors had a positive influence on recruitment, only SPrec was significant (Table 4), indicating that the recruitment of new individuals increases during the rainy season. There was also a significant negative interaction between these predictors on the recruitment of new seedlings, demonstrating that the effect of the rainy season precipitation decreased as time elapsed or the effect of time decreased as rainfall increased. Fences did not affect the rate of recruitment.

## Discussion

### State of natural regeneration

Seasonality had a large influence on the number of individuals throughout the study period, with the abundance increasing during the rainy season and decreasing during the dry season, as would be expected (Fig. 1a). This difference was particularly evident in the unfenced plots. By contrast, Rodriguez^28^ found that the number of individuals decreased over time in both unfenced and fenced plots in a smaller part of the same study area, even during the rainy season. Our finding that there was an almost constant number of individuals in the fenced plots showed that a fencing effect mainly occurred during the dry season, when food availability was lower and animals needed to be meticulous in searching for food. Similarly, in an evaluation of the effects of ungulates (cattle and goats) in Hawaii, Cabin *et al*.^33^ found that there was a much higher abundance of seedlings in a year that was catalogued as being rainy (1997) compared with a year that was exceptionally dry (1998), when there was a marked decrease in the number of seedlings across both treatments and no additional individuals of the most abundant species [*Diospyros sandwicensis* (A.DC.) Fosberg] throughout the year.

Previous studies have also found that mortality is highly seasonal^28,33,34^. For example, Rodriguez^28^ reported maximum mortalities of 20% in the rainy season and 67% in the dry season (across 4 and 2 months, respectively), and Lieberman & Li^34^ reported maximum mortalities of 12% in the rainy season and 33% in the dry season (across 2 months in both cases). Similarly, in the present study, we obtained maximum mortality values of 23% for the rainy season and 85% for the dry season (across 4 and 6 months, respectively). If we make the same comparison for the fenced plots, the maximum values were 23% for the rainy season and 36% for the dry season (across 2 months for each) in the study of Rodriguez^28^ and 35% in the rainy season and 87.5% in the dry season (across 4 and 6 months, respectively) in the present study. These findings indicate that mortality during the dry season was much higher in the present study than has previously been reported, which may have resulted from the low number of individuals in some samples resulting in most or all of the seedlings (e.g. two from a total of three seedlings) dying before the next evaluation.

Lieberman & Li^34^, Cabin *et al*.^33^ and Vieira & Scariot^35^ stated that both mortality and recruitment are highly seasonal in tropical dry forests, with higher mortality in the dry season and higher recruitment in the rainy season. By contrast, we found that there was a continuous decrease in recruitment in the unfenced plots, even during the rainy season (Fig. 1c). However, recruitment in the fenced plots was closer to their prediction – for example, there was similar recruitment between the first dry period and the next wet period, followed by a sharp decrease in the second dry period (Fig. 1c). The unclear seasonal influence in our study area could be explained by the unusual climatic situation during the previous rainy season (survey 2), when precipitation was restricted to only two or three relevant events in January, almost no rainfall in February and a short rainy period from March to May, followed by some light showers even in June, which contrasts with the continuous rains that usually occur from December to March. Therefore, it is likely that several seedlings that established during the wet period (after our assessment) were recorded in the dry period in survey 3.

It has previously been noted that seed banks in tropical dry forests contain low numbers of seeds and species^18,36^. Ceccon *et al*.^36^ and Ray & Brown^37^ argued that the low number of species with high seed germination rates and high mortality rates was mainly caused by environmental stress, predation and dispersal restrictions and, according to Espinosa *et al*.^18^, our study area is also affected by these conditions. Therefore, since the low availability of seeds negatively affects the species richness and abundance, shoot propagation is considered more important than seed propagation in the study area^18,30^.

The total number of species we recorded was considerably higher than the 21 species reported by Aguirre *et al*.^30^ for the Ecuadorian part of the same study area and the 53 species reported by Lieberman & Li^34^ in a small 120-ha tropical dry forest patch located in Ghana. As in the present study, Rodriguez^28^ detected a small difference in species richness between treatments, with a slightly higher number of species and diversity index in fenced plots. However, unlike our results, Rodriguez^28^ also found that the number of species decreased over time in fenced plots, which could be related to the fact that recruitment was not accounted for. Similarly, Cabin *et al*.^33^ found that plots in a preserved area that had been fenced for 40 years had a larger number of species than unfenced plots, as well as a larger number of native than exotic species.

Species that were characteristic of the study area^32^, such as *Ceiba trischistandra*, *Cavanillesia platanifolia*, *Eriotheca roseorum* and *Myroxylon balsamum*, were previously recorded in a mature forest inventory^26^ and, according to Aguirre & Kvist^20^, populations of the first two of these species have been maintained or may even have recovered. However, we did not record seedlings of these species in our study, and seedlings of *Centrolobium ochroxylum* and *Loxopterygium huasango* were scarce. This situation was also pointed out by Aguirre *et al*.^30^, who attributed it to a few species having abundant regeneration in dry forests and strong perturbations. However, it may also be caused by the low diversity in the seed bank in this region^18^ or the presence of selective seed or seedling predators.

### Predictors affecting the natural regeneration

One factor that has been shown to determine the establishment, survival and development of natural regeneration in dry forest is the availability of water^18,38^. Similarly, in the present study, seasonal precipitation (the rainy season) had the highest positive influence on all of the parameters analysed except diversity, with the abundance of seedlings, the number of species and recruitment increasing significantly and mortality decreasing significantly during the rainy season (Tables 2 and 4). Similar results were also presented by Espinosa *et al*.^18^, Lieberman & Li^34^, Cabin *et al*.^33^ and Vieira & Scariot^35^. These effects can be attributed to the higher water availability, the accelerated decomposition of organic matter^18^, and the higher concentration and uptake of nutrients that accumulate in the soil through the dry season when the uptake by vegetation is lower^39^.

It is well known that unusual events such as El Niño bring major changes in the atmosphere, with temperatures that can exceed the normal average by 2 °C, while the rainfall over Peru and Ecuador can greatly increase the vegetation cover of dry lands^40^. Therefore, a longer assessment could allow the effects of these events on natural regeneration to be analysed.

Although treatment was included in the best models for all of the parameters except diversity, fencing only had a significant positive effect on mortality and by interaction with Equine on abundance. By contrast, Rodriguez^28^ and Cabin *et al*.^33^ found a meaningful difference between unfenced and fenced samples for aspects such as species richness and abundance. In the present study, species richness and recruitment exhibited only small differences between fenced and unfenced plots (Tables 2 and 4). Furthermore, although well-established young individuals between 2–3 m of height were found on private land with longer exclusion times (approximately 6 years), these were not recorded in any of our plots and only a few have been reported in the study area^30^. Therefore, we believe that our observation time of 2 years may not have been sufficient to reveal direct fencing effects.

Fences were found to be associated with decreased mortality (Table 3), which is consistent with the findings of Rodriguez^28^. This is because fences reduce the impact of animals that eat and trample seedlings^20^ and of people that damage individual plants when they walk through the forests or carry out extractive activities.

Animals affected only the abundance of seedlings, with cattle having significant negative effects and horses and donkeys having significant positive effects (Table 2). This difference may have been caused by the current numbers of these animals in the area. It has previously been shown that cattle exert a negative effect on the structure of mature forests, particularly in terms of the abundance of individuals^41–43^, but there is little information about the effects of horses or donkeys on mature and young plants. Since these groups of animals have similar physical traits and food preferences, we believe that they are highly likely to have a similar impact on the forest, suggesting that the number of animals is a critical factor for the level of impact, i.e. a high number of horses or cows would lead to a high impact. Horses and donkeys were once an essential means of transport, but this function has decreased since the introduction of motorcycles. By contrast, cattle are used for meat, milk or sale, leading to a considerable increase in number (Fig. 2f). The reduced number of horses could have favoured regeneration through the contribution of manure, the scarification or distribution of seeds, reduced trampling and the low number of seedlings being eaten. When considering these results, it is important to note that our evaluation of the influence of animals was based on faecal samples, which decompose following exposure to environmental conditions such as sun, wind and water. Therefore, the effects of animals should be interpreted with caution.

Wild animals could also affect natural regeneration as well as leaf litter covering the seedlings. However, they were not considered in this study, because of the difficulty to obtain suitable data and the limited budget.

Soil depth and drainage were important predictors of species richness, with soils that were >10 cm deep and better drained hosting larger numbers of species (Table 2). Alban *et al*.^44^ previously reported that *Prosopis pallida* seeds that were sown superficially (5 cm depth) were damaged or eaten by ants and lizards, and surface seeds will also be accessible to selective birds and rodents. Furthermore, nutrients are transported by leaching rainwater towards deeper soils^39^, leading to a better nutritive status of deeper seeds compared to those at the surface. Since this leaching will depend on the soil drainage capacity, the lower species richness and diversity that occurs in poorly drained soils can also be explained by lower nutrient movement into the soil.

Soil texture was a significant predictor of diversity, with clay loam and loam soils having higher levels of diversity than clay–sandy loam soils (Table 2). This can be explained by the high nutrient and water storage capacity, good aeration, and good root penetration of loam soils compared with sandy soils, all of which favour germination and plant development^45^.

Elapsed time was an important predictor of mortality and recruitment (Table 3). Similarly, Rodriguez^28^ showed that mortality exhibited a constant decrease over time that was disrupted only by the rainy season. This effect was expected because the number of individuals decreases throughout the dry season and the surviving individuals will have adapted to those conditions, thus decreasing mortality.

In contrast to our findings in the mature forest^26^, there was no evidence that the human pressure index (HPI) affected natural regeneration. One likely reason for this is that fencing restricted the activities of humans as well as animals within the plots. We also found that there was a high correlation between HPI and 10 of the other 13 predictors we used [all except SPrec, Treat and stoniness (Ston); see Supplementary Table S2], which may have limited the inclusion of this predictor in the models.

Lieberman & Li^34^ identified canopy closure as an important predictor of forest density. However, as for HPI, canopy closure was highly correlated with several of the predictors considered in our study and was considered in only 12 models (see Supplementary Table S3). However, we found that both the number of species and the abundance of individuals tended to increase with canopy closure. This would be expected if we consider that a closed canopy favours seed germination and seedling growth because it helps to prevent the seeds of species that are dispersed by wind and gravity, which are usually produced in the dry season^46,47^, from becoming desiccated by high temperatures. Furthermore, a closed canopy also helps to prevent fresh fruits and seeds that are dispersed by animals, which mainly occur in the rainy season^47^, from being washed away during heavy rainfall.

## Conclusions

Seasonal precipitation is a highly influential predictor of natural regeneration in the dry forest. However, unfortunately, the rainy season is also the period with the greatest pressures due to the high availability of food for animals. Therefore, suitable management strategies should be introduced to help halt the loss of abundance and richness due to aspects not related to drought. These programmes could be coordinated by local government and supported by farmers’ organisations and communities and could include compensation for farmers for losses during the temporary closure of certain areas for 5 years or more.

Since an almost constant species richness was recorded during the 2 years of assessment, we assume that this parameter is not affected by anthropogenic activities or abiotic factors in our study area in the medium term. Furthermore, a substantial number of seedlings was recorded during the rainy period. However, more accurate information on the mortality, recruitment and abundance of individuals during the distinct phases of regeneration is essential for determining whether the forest structure will be affected in the near future.

While the use of fences to exclude all types of activity is important in the study area, this will not affect the diversity and structure of the future forest if they are only established for a short period of time. Therefore, fences must be maintained on a medium- to long-term basis.

Cattle currently deserve special attention, as they are affecting the abundance of seedlings. Control measures have already been introduced at some sites in the study area, such as regulating the number of cows per family that are allowed in the forest. However, this research shows that this rule is not working either because it is not being complied with or because the proposed limit is still too high. Since our approach of estimating the intensity of animal impact from faeces has several weaknesses, more precise animal inventories are recommended in the future, as well as determination of the carrying capacity of the forest.

Soil conditions have a much larger effect on natural regeneration than on the mature forest in the study area. Texture, drainage and soil depth are all important predictors that can generate changes in the species richness and diversity of natural regeneration. Thus, areas with suitable conditions should be identified, fenced and used as seedling production sites.

Finally, our results indicate that mortality is vulnerable to both seasonality and the actions of animals and humans, whereas recruitment is mostly affected by the former.

## Methods

### Study area

**Figure 3:**
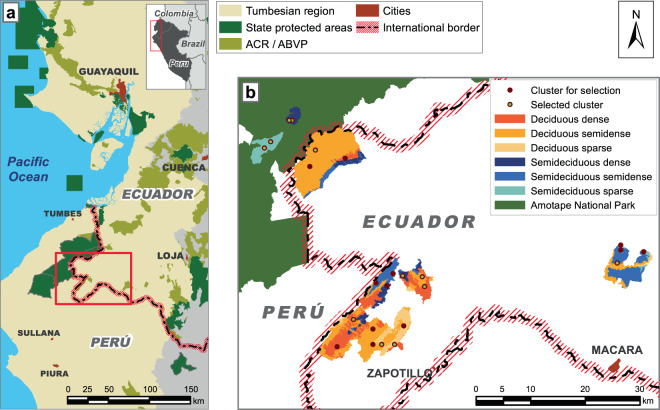
Geographic location of the study area. The maps were built using QGIS Desktop v. 2.8.7^76^ (http://qgis.osgeo.org) (**a**) Locations of the study area and protected areas within the Tumbesian region. ‘ACR/ABVP’, areas of regional conservancy of Perú and area of protective forests and other vegetation of Ecuador. Modified from *“La Región Tumbesina–una riqueza compartida*^77^*”* **(b)** Distribution of clusters in the study area.

This research was conducted in the central part of the Tumbesian region, in southwestern Ecuador and north-western Peru (Fig. 3a). This area ranges from 200 to 1,100 m above sea level (a.s.l.)^48,49^ and has a mean annual temperature of 20–26 °C, although this can exceed 35 °C during the rainy season^50^. The annual precipitation ranges from 300 to 700 mm^20^, but nearly 85% of this is registered in the 4 months from January to April and there are two or three critically dry months (September–November). Consequently, two seasons can be clearly identified according to the presence or absence of rainwater. The region is affected by El Niño events, which occur every 3–7 years and bring intense rains that cause a change in the climate^40^.

Colonisation of the area dates back to the pre-Columbian civilisations^51^, which means that there has been quite a long period of intervention in the dry forest ecosystem, with increasingly harmful management practices being undertaken. This induced a shift in the landscape until it reached its current state. A period of intensive logging of *Handroanthus chrysanthus* (Jacq.) S.O.Grose and *H. billbergii* (Bureau & K.Schum.) S.O.Grose that had occurred for almost two decades was stopped in 1978 through the declaration of ‘closed areas’ in lands below 1,000 m a.s.l in southwest Ecuador^52^. However, timber extraction for domestic use (generally of selected species) continues today because it is a permitted activity on state lands^52^, and illegal extraction has not been completely eliminated in either country^20,50^. Furthermore, livestock management is deficient or inexistent, with goats, cattle, horses and donkeys being released to graze freely in the forest.

### Data collection

#### Natural regeneration

Based on the dry forest types described by Lozano^53^ and Aguirre *et al*.^49^, we considered two formations (deciduous and semi-deciduous) and following Cueva & Chalán^54^ three density levels (dense, semi-dense and sparse). This resulted in six different types of forest, which were considered to adequately cover most of the forest variability.

**Figure 4:**
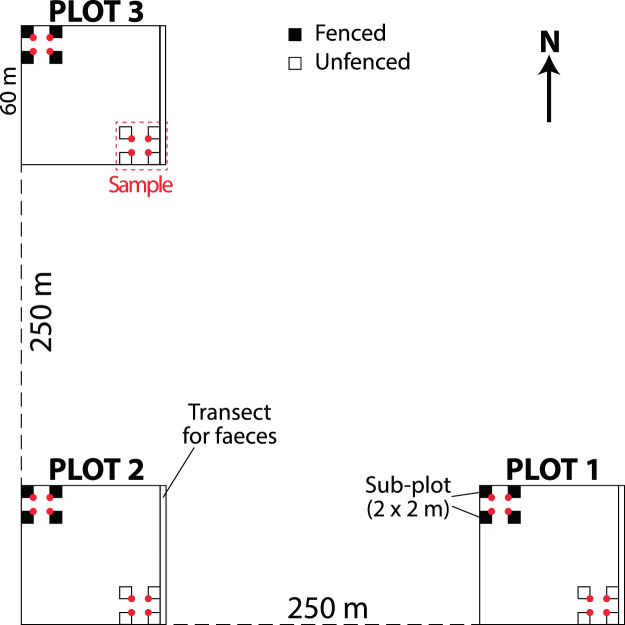
Diagram of the cluster design. Red dots indicate the places where photographs of the canopy were captured. Transects along the eastern edges (1 × 60 m) were used to collect faeces.

Four clusters were installed in each type of forest to give 24 clusters in total (Fig. 3b). Each of these clusters comprised three 60 × 60 m plots, within of which there were four 2 × 2 m sub-plots. After the first regeneration inventory, 12 clusters were selected from a matrix of high, medium and low regeneration abundances and species diversities. Additional sub-plots were then installed and fenced in each of these 12 clusters (four per large plot) (Fig. 4) to give four unfenced and four fenced sub-plots per plot (or 288 sub-plots in total: 144 fenced and 144 unfenced). Since the distance between the sub-plots was only 16 m and no variability was perceptible, they were grouped and treated as a single sample (Fig. 4). Thus, 12 clusters (8 in Ecuador and 4 in Peru) containing 72 samples (36 per treatment) were assessed over a total area of 1,152 m^2^.

Over the almost 2-year study period, five surveys of the unfenced plots were carried out in Dec. 2014, Jul. 2015, Jan. 2016, May 2016 and Nov. 2016, and four surveys of the fenced plots were carried out at the same times with the exception of Dec. 2014. During each survey, all of the seedlings of trees and shrubs that were >5 cm tall were identified and recorded. In addition, from survey 2 onwards, any dead seedlings were recorded and all live seedlings were labelled. Thus, mortality and recruitment were computed from survey 3 onwards.

#### Biotic and abiotic factors

To estimate the number of grazing animals, in the first survey the faeces of goats, cows, horses and donkeys were collected along a 1 m × 60 m transect placed along the eastern border of the larger plots (Fig. 4) and separated according to the animal group. The dry weights of these samples were then obtained, combining the values for horses and donkeys.

To estimate the intensity of general anthropogenic pressure caused by activities such as firewood or timber extraction, trampling, grazing, etc., Hegyi’s competition index was adapted^55^, using the expression defined in Cueva *et al*.^26^ to compute the HPI.

To characterise the light conditions in the plots, photographs of the canopy (Canp) were captured with a Canon EOS 300D DIGITAL camera using an 8-mm fisheye lens. One photograph was taken at the internal corners of each 2 × 2 m sub-plot (Fig. 4) in both the rainy and dry seasons. The camera was always placed 1.30 m above the ground facing north.

We included the available abiotic variables that are considered to have high influence on the structure and species composition of the tropical dry forest ecosystem^1,2^.

Climatic information gridded to a resolution of 30 arc-seconds (approximately 1 km^2^) was obtained from Fick & Hijmans^31^. The seasonal precipitation (SPrec) was obtained by summing separately the rainfall from January to April to obtain the amount of precipitation in the rainy season and the rainfall from May to December to obtain the amount of rainfall in the dry season. The mean annual temperature (MTemp) was obtained as an average. The altitude (Alt) was measured in the field for each plot.

For the Ecuadorian part of the study area, soil data were obtained from the Geopedological Map of Zapotillo and Celica of the Instituto Espacial Ecuatoriano^56^. The soil parameters used were organic material (OM), soil depth (SDepth), drainage (Drain), stoniness (Ston) and texture (Text). For the Peruvian part of the study area, the Soil Classification Map of the Oficina Nacional de Evaluación de Recursos Naturales (ONERN)^57^ was used to identify the soil type. Unfortunately, this work does not include specific information about the soil characteristics, so they were assumed to be identical to those of the same soil type in the nearest localities on the Ecuadorian side.

Time was considered as the number of elapsed months since the first survey. Treatment (Treat) comprised two groups: open for unfenced plots and exclusion for fenced plots.

The ranges or levels of all predictors can be found in Supplementary Table S4, the full database used to evaluate structure and diversity is provided in the Supplementary Table S5 and the dataset to evaluate dynamics in the Supplementary Table S6.

### Data analysis

#### Data processing

Abundance (Ind) and species richness (Spp) were calculated by counting the number of live individuals and species, respectively, of tree and shrub seedlings in each sample, treatment and survey period. Mortality (Mort) was computed by considering the percentage of dead individuals in relation to the number of individuals in the previous survey. The recruitment rate (Recr) was obtained by calculating the percentage of new individuals in a survey in relation to the total number of individuals in the same survey. Diversity was computed by calculating Simpson’s index (Simp), as this is considered an effective and robust diversity measure^58^.

The canopy images were processed by Geigl^59^ using Gap Light Analyzer v2.0 software^60^ (available on https://www.caryinstitute.org/science/our-scientists/dr-charles-d-canham/gap-light-analyzer-gla). Canopy coverage was computed as a percentage, and the average among the four sub-plots (unfenced and fenced plots were averaged separately) was used for each sample.

#### Statistical analysis

Generalized linear mixed models (GLMMs)^61–63^ were applied to identify the effects of various predictors on the parameters evaluated. The response variables were the abundance of regeneration as a structure indicator; mortality and recruitment as indicators of the dynamics; and species richness and Simpson’s index as indicators of the diversity of natural regeneration.

A total of 14 predictors were used: five biotic indicators (Goats, Cattle, Equine, HPI and Canp); eight abiotic variables related to climate, geography and soil (SPrec, MTemp, Alt, OM, SDepth, Drain, Ston and Text); and treatment (Treat). To improve the model fitting the predictor variables were transformed as shown in the Supplementary Table S4.

Correlations among all of the predictor variables were tested (Supplementary Table S2) and models were then built by considering that strongly correlated predictors could not be included in the same model to avoid collinearity. In addition, a maximum of five predictors were included in each model to avoid overfitting^64^, resulting in 168 candidate models. A further 18 models were also built that included interactions between variables representing anthropogenic pressure and those that Cueva *et al*.^26^ identified as having a relevant influence on the structure and diversity of the mature forest. Thus, a total of 186 candidate models were tested (see Supplementary Table S3). The asterisk (*) between interaction terms represents both the higher-order interaction term as well as the lower-order main effects.

The predictors SPrec, Treat and Time and their interactions were chosen to predict mortality and recruitment. In this instance, we only evaluated the effect of these three predictors because our goal was to assess if animals are more influential than seasonality, which would allow us to say if management policies are required to improve conditions in the dynamic of natural regeneration. Thirteen models were built for this purpose (see Supplementary Table S7). In all cases, formation and cluster were considered as random effects (plots were nested within clusters, and clusters were nested within formations), and Time was considered as a random slope to account for the repeated measures.

The effects of the predictors on abundance were assessed using the maximum likelihood approach with a negative binomial error distribution to deal with overdispersion^65^. The effects of the predictors on species richness were assessed using a GLMM with a Poisson error distribution and the Laplace approximation to get a true likelihood^62,66^. The effects of the predictors on diversity were assessed using the REML approach^62^. All of these analyses were performed using the lme4 package^61^ v1.1–21. The effects on mortality were assessed using the REML approach and Bayesian fitting to deal with singular fitting^67^ using the blme package^68^ v1.0–4. Finally, the effects of the predictors on recruitment were assessed using a GLMM with a Gaussian error distribution and link identity because 22% of the recruitment values were zeros. The glmmTMB package^69^ was used, as this is able to deal with zero-inflated model^70^.

Model selection was carried out using the delta Akaike information criterion (∆AIC < 2)^71^. For all models, we computed the marginal and conditional variance (*R2m* and *R2c*, respectively)^72^ to determine the proportion of the variance that was explained by each model. *R2m* and *R2c* were calculated using the MuMIn package^73^ v1.42.1 when the lme4 and blme packages were used and the sjstats package^74^ v0.17.4 when the glmmTMB package was used.

All analyses were performed in the R programming environment v3.5.3^75^.

## Supplementary information

Supplementary information.

## Data Availability

The datasets are available as supplementary information. R scripts available upon request. Please email to jorge.cueva@tum.de for R scripts derived from freely available R packages: see material and methods for more details.

## References

[1] Janzen, D. H. Tropical dry forests: The most endangered major tropical ecosystem. *Biodiversity*, 10.17226/989 (National Academy Press 1988).

[2] Gentry AH (1988). Changes in Plant Community Diversity and Floristic Composition on Environmental and Geographical Gradients. Ann. Missouri Bot. Gard..

[3] Best, B. J. & Kessler, M. *Biodiversity and Conservation in Tumbesian Ecuador and Peru*. (BirdLife International, 1995).

[4] Olson, D. M. *et al*. *The Global 200: A Representation Approach to Conserving the Earth’s Distinctive Ecoregions*. (2000).

[5] Miles L (2006). A global overview of the conservation status of tropical dry forests. J. Biogeogr..

[6] Semper-Pascual A (2018). Mapping extinction debt highlights conservation opportunities for birds and mammals in the South American Chaco. J. Appl. Ecol..

[7] Myers N, Mittermier RA, Mittermier CG, da Fonseca GA, Kent J (2000). Biodiversity hotspots for conservation priorities. Nature.

[8] Linares-Palomino, R., Oliveira-Filho, A. & Pennington, R. T. Neotropical Seasonally Dry Forests: Diversity, Endemism, and Biogeography of Woody Plants. *in Seasonally Dry Tropical Forests: Ecology and Conservation* (eds. Dirzo, R., Young, H. S., Mooney, H. A. & Ceballos, G.) 3–21, 10.5822/978-1-61091-021-7 (Island Press 2011).

[9] Banda-R. K (2016). Plant diversity patterns in neotropical dry forests and their conservation implications. Science (80-.)..

[10] Ministério do Meio Ambiente. Biodiversidade Brasileira: Avaliação e identificação de áreas e ações prioritárias para conservação, utilização sustentável e repartição dos benefícios da biodiversidade nos biomas brasileiros. http://www.icmbio.gov.br/revistaeletronica/index.php/BioBR/article/view/140/115 (2002).

[11] Koleff P, Urquiza-Haas T, Contreras B (2012). Prioridades de conservación de los bosques tropicales en México: reflexiones sobre su estado de conservación y manejo. Ecosistemas.

[12] UNESCO. Bosques de Paz Transboundary Biosphere Reserve (Ecuador/Peru). *UNESCO*, https://en.unesco.org/biosphere/transboundary/bosques-de-paz (2017).

[13] Ministerio del Ambiente del Perú. *Decreto Supremo que establece el Área de Conservación Regional Bosques Tropicales Estacionalmente Secos del Marañón*. 34–38 (El Peruano, 2018).

[14] Blackie, R. *et al*. Tropical dry forests: The state of global knowledge and recommendations for future research. *CIFOR* **30**, 10.17528/cifor/004408 (2014).

[15] Sunderland T (2015). Global dry forests: a prologue. Int. For. Rev..

[16] Stoner KE, Sánchez-Azofeifa GA (2009). Ecology and regeneration of tropical dry forests in the Americas: Implications for management. For. Ecol. Manage..

[17] Dinerstein, E. *et al*. *Una Evaluación del estado de conservación de las eco-regiones terrestres de América Latina y el Caribe*. (Banco Internacional de Reconstrucción y Fomento/Banco Mundial, 1995).

[18] Espinosa CI, De La Cruz M, Luzuriaga L, Escudero A (2012). Bosques tropicales secos de la región pacífico ecuatorial: diversidad, estructura, funcionamiento e implicaciones para la conservación. Ecosistemas.

[19] Linares-Palomino R, Kvist LP, Aguirre MZ, Gonzales-Inca C (2009). Diversity and endemism of woody plant species in the Equatorial Pacific seasonally dry forests. Biodivers. Conserv..

[20] Aguirre MZ, Kvist LP (2005). Floristic composition and conservation status of the dry forests in Ecuador. Lyonia.

[21] Aguirre MZ, Geada-Lopez G (2017). Estado de conservación de los bosques secos de la provincia de Loja, Ecuador Conservation status of the dry forests of the province of Loja. Arnaldoa.

[22] Jara-Guerrero A, De la Cruz M, Méndez M (2011). Seed Dispersal Spectrum of Woody Species in South Ecuadorian Dry Forests: Environmental Correlates and the Effect of Considering Species Abundance. Biotropica.

[23] Espinosa, C. I. Estructura y funcionamiento de ecosistemas secos del Sur de Ecuador. *Tesis Doctoral* (Universidad Politécnica de Madrid, 2012).

[24] Espinosa CI, Cabrera O, Luzuriaga A, Escudero A (2011). What factors affect diversity and species composition of endangered tumbesian dry forests in southern Ecuador?. Biotropica.

[25] Piana RP, Marsden SJ (2014). Impacts of cattle grazing on forest structure and raptor distribution within a neotropical protected area. Biodivers. Conserv..

[26] Cueva OJ (2019). Influence of anthropogenic factors on the diversity and structure of a dry forest in the central part of the Tumbesian region (Ecuador-Perú). Forests.

[27] Castro MG, Tigabu M, González RB, Odén PC (2009). Natural regeneration dynamics of three dry deciduous forest species in Chacocente Wildlife Reserve, Nicaragua. J. For. Res..

[28] Rodriguez, M. T. *Influencia del ganado caprino en el sotobosque del ecosistema bosque seco de la comunidad Cabeza de Toro-cantón Zapotillo (Loja-Ecuador)*. (Universidad del Azuay, 2006).

[29] Abou Rajab, Y. *et al*. Stand structure, natural regeneration and tree growth in the seasonally dry forest of El Angolo, *Northern Peru*. (University of Hohenheim, Universidad Nacional Agraria La Molina, Forest Ecology and Remote Sensing, 2010).

[30] Aguirre MZ, Betancourt Figueras Y, Geada López G (2013). Regeneración natural en los bosques secos de la provincia de Loja y utilidad para el manejo local. Cedamaz.

[31] Fick SE, Hijmans RJ (2017). WorldClim 2: new 1-km spatial resolution climate surfaces for global land areas. Int. J. Climatol..

[32] Aguirre MZ, Kvist LP (2009). Composición florística y estructura de bosques estacionalmente secos en el sur-occidental de Ecuador, provincia de Loja, municipios de Macara y Zapotillo. Arnaldoa.

[33] Cabin RJ (2000). Effects of Long‐Term Ungulate Exclusion and Recent Alien Species Control on the Preservation and Restoration of a Hawaiian Tropical Dry Forest. Conserv. Biol..

[34] Lieberman D, Li M (1992). Seedling Recruitment Patterns in a Tropical Dry Forest in Ghana. J. Veg. Sci..

[35] Vieira DLM, Scariot A (2006). Principles of natural regeneration of tropical dry forests for restoration. Restor. Ecol..

[36] Ceccon E, Huante P, Rincón E (2006). Abiotic factors influencing tropical dry forests regeneration. Brazilian Arch. Biol. Technol..

[37] Ray GJ, Brown BJ (1994). Seed Ecology of Woody Species in a Caribbean Dry Forest. Restor. Ecol..

[38] Ruthenberg, H. *Farming Systems in the Tropics*. (Clarendon Press, 1971).

[39] Roy S, Singh JS (1995). Seasonal and Spatial Dynamics of Plant-Available N and P Pools and N-Mineralization in Relation to Fine Roots in a Dry Tropical Forest Habitat. Soil Biol. Biochem..

[40] Trenberth, K. E. El Niño Southern Oscillation (ENSO). In *Encyclopedia of Ocean Sciences* (eds. Cochran, J. K., Bokuniewicz, H. J. & Yager, P. L.) 420–432, 10.1016/B978-0-12-409548-9.04082-3 (Elsevier Inc. 2019).

[41] Stern M, Quesada M, Stoner KE (2002). Changes in composition and structure of a tropical dry forest following intermittent cattle grazing. Rev. Biol. Trop..

[42] Cierjacks A, Hensen I (2004). Variation of Stand Structure and Regeneration of Mediterranean Holm Oak along a Grazing Intensity Gradient along a grazing intensity gradient. Plant Ecol..

[43] Schulz K (2016). Grazing deteriorates the soil carbon stocks of Caatinga forest ecosystems in Brazil. For. Ecol. Manage..

[44] Albán L, Matorel M, Trías J, Vera J (2002). Reforestación extensiva con algarrobo (Prosopis pallida) en la Región Desértica de Piura. Perú. Rev. Zo. áridas.

[45] Andrades, M. & Martínez, M. E. Fertilidad del suelo y parámetros que la definen. Agricultura y alimentación (Publication service, Universidad de la Rioja, 2014).

[46] Bullock, S. H. Plant reproduction in neotropical dry forests. In *Seasonally Dry Tropical Forests* (eds. Bullock, S. H., Mooney, H. A. & Medina, E.) 277–303, 10.1017/CBO9780511753398.011 (Cambridge University Press, 1995).

[47] Griz MLS, Machado IC (2001). Fruiting phenology and seed dispersal syndromes in Caatinga, a tropical dry forest in the northeast of Brazil. J. Trop. Ecol..

[48] Gentry, A. H. Diversity and floristic composition of neotropical dry forests. In *Seasonally Dry Tropical Forests* (eds. Bullock, S. H., Mooney, H. A. & Medina, E.) 146–194 (Cambridge University Press, 1995).

[49] Aguirre M. Z., Kvist, L. P. & Sánchez, T. O. Bosques secos en Ecuador y su diversidad. In *Botánica Económica de los Andes Centrales* (eds. Morales R., M., Øllgaard, B., Kvist, L. P., Borchsenius, F. & Balslev, H.) 162–187 (Universidad Mayor de San Andrés, 2006).

[50] Leal-Pinedo JM, Linares-Palomino R (2005). Los bosques secos de la reserva de biosfera del noroeste (Perú): Diversidad arbórea y estado de conservación. Caldasia.

[51] Hocquenghem, A. M. Para Vencer la Muerte: Piura y Tumbes. Raíces en el Bosque Seco y en la Selva Alta—Horizontes en el Pacífico y en la Amazonia. (Centro Nacional de la Investigación Científica (CNRS)/Instituto Francés de Estudios Andinos (IFEA)/Instituto de la Naturaleza y el Conocimiento Ambiental Humano (INCAH), 1998).

[52] Ministerio de Agricultura y Ganadería, Ministerio de Industrias Comercio e Integración & Ministerio de Defensa Nacional. *Declaratoria de veda total a la explotación forestal en los bosques de las provincias de Loja y El Oro*. Registro Oficial 595-0162 3–4 (1978).

[53] Lozano C. P. E. Los tipos de bosque en el sur del Ecuador. In *Botánica Austroecuatoriana: estudios sobre los recursos vegetales en las provincias de El Oro, Loja y Zamora-Chinchipe* (eds. Aguirre M., Z., Madsen, J. E., Cotton, E. & Balslev, H.) 29–49 (Abya Yala, 2002).

[54] Cueva O. J. Elaboración y análisis del estado de la cobertura vegetal de la provincia de Loja - Ecuador. (Universidad Internacional de Andalucía, 2012).

[55] Hegyi, F. A simulation model for managing Jack-pine stands. in Growth models for tree and stand simulation (ed. Fries., J.) 74–90 (Royal College of Forestry, 1974).

[56] Instituto Espacial Ecuatoriano. Mapa geopedologico de Celica y Zapotillo. IDE-PORTAL http://www.ideportal.iee.gob.ec (2018).

[57] Oficina Nacional de Evaluación de Recursos Naturales (ONERN). *Mapa de Suelos*. https://www.arcgis.com/home/webmap/viewer.html?webmap=8eb6a604e5b042bfb41e6373699d2054 (2015).

[58] Magurran, A. E. *Measuring Biological Diversity*. (Blackwell Publishing 2004).

[59] Geigl, M. J. *Hemisphärische Aufnahmen der Überschirmung zur Analyse der Entwicklung der Naturverjüngung im tumbesischen Trockenwald*. (Technische Universität München, 2018).

[60] Frazer, G. W., Canham, C. D. & Lertzman, K. P. Gap Light Analyzer: Imaging software to extract canopy structure and gap light transmission indices from true-colour fisheye photographs. Users Manual and Program Documentation, Version 2.0. https://www.caryinstitute.org/science/our-scientists/dr-charles-d-canham/gap-light-analyzer-gla (1999).

[61] Bates DM, Mächler M, Bolker BM, Walker SC (2015). Fitting Linear Mixed-Effects Models using lme4. J. Stat. Softw..

[62] Bolker BM (2009). Generalized linear mixed models: a practical guide for ecology and evolution. Trends Ecol. Evol..

[63] Cayuela, L. Modelos lineales mixtos (LMM) y modelos lineales generalizados mixtos (GLMM) en R. *Curso de análisis de datos ecológicos en R* (2014).

[64] Neo L, Yee ATK, Chong KY, Kee CY, Tan HTW (2017). Vascular plant species richness and composition in two types of post-cultivation tropical secondary forest. Appl. Veg. Sci..

[65] Di Rienzo, J. A., Macchiavelli, R. & Casanoves, F. Modelos lineales generalizados mixtos: aplicaciones en InfoStat. 101 http://repositorio.bibliotecaorton.catie.ac.cr/handle/11554/8691?show=full (2017).

[66] Bolker, B. M. *et al*. GLMMs in action: gene-by-environment interaction in total fruit production of wild populations of Arabidopsis thaliana Revised version, part 1. 1–33 http://glmm.wdfiles.com/local–files/examples/Banta_2011_part1.pdf (2011).

[67] Bolker, B. M. GLMM FAQ. https://bbolker.github.io/mixedmodels-misc/glmmFAQ.html#singular-models-random-effect-variances-estimated-as-zero-or-correlations-estimated-as–1 (2019).

[68] Chung Y, Rabe-Hesketh S, Dorie V, Gelman A, Liu J (2013). A nondegenerate penalized likelihood estimator for variance parameters in multilevel models. Psychometrika.

[69] Brooks ME (2017). glmmTMB balances speed and flexibility among packages for zero-inflated generalized linear mixed modeling. R J..

[70] Bolker, B. M. Getting started with the glmmTMB package. **9** https://cran.r-project.org/web/packages/glmmTMB/vignettes/glmmTMB.pdf (2019).

[71] Burnham, K. P. & Anderson, D. R. Model selection and multimodel inference: a practical information-theoretic approach. (Springer, 2002).

[72] Nakagawa, S. & Schielzeth, H. A general and simple method for obtaining R^2^ from generalized linear mixed-effects models. *Methods Ecol. Evol.* **4**, 133–142 (2013).

[73] Bartoń, K. MuMIn: Multi-model inference. CRAM https://cran.r-project.org/web/packages/MuMIn/index.html (2018).

[74] Lüdecke, D. sjstats: Statistical Functions for Regression Models. *Zenodo*, 10.5281/zenodo.1284472 (2019).

[75] R Core Team. *R: A language and environment for statistical computing*. https://www.r-project.org/ (2019).

[76] QGIS Development Team. *QGIS Geographic Information System*. http://qgis.osgeo.org (2009).

[77] Cueva O., J. & Rodas, F. *La**Región Tumbesina: una riqueza compartida*. (Naturaleza & Cultura Internacional, 2010).

