# Supplementary material for: Natural Regeneration in the Tumbesian Dry Forest: Identification of the Drivers Affecting Abundance and Diversity
Source: Sci Rep. 2020 Jun 17;10:9786. doi: 10.1038/s41598-020-66743-x (PMC7299929; doi:10.1038/s41598-020-66743-x)
Supplement: Supplementary file 1 — Supplementary information. [file 41598_2020_66743_MOESM1_ESM.pdf]

Supplementary Information

Manuscript: Natural Regeneration in the Tumbesian Dry Forest: Identification of the Drivers Affecting Abundance and Diversity.

Authors: Jorge Cueva Ortiz[1,*], Carlos Iván Espinosa[2], Zhofre Aguirre Mendoza[3], Elizabeth Gusmán-Montalván[2], Michael Weber[1] & Patrick Hildebrandt[1]

1. Institute of Silviculture, TUM School of Life Sciences Weihenstephan, Technical University of Munich, Freising, 85354, Germany.
2. EcoSs_Lab, Departamento de Ciencias Biológicas, Universidad Técnica Particular de Loja, San Cayetano Alto, Loja, 110107, Ecuador.
3. Carrera de Ingeniería Forestal, Universidad Nacional de Loja, Ciudadela Guillermo Falconí E., Loja, 110-110, Ecuador.

* jorge.cueva@tum.de; Tel.: +49-8161-71-4690

Supplementary Table S1. Trees and shrubs species of natural regeneration in the study area

Family	Species
Achatocarpaceae	*Achatocarpus pubescens* C. H. Wright
Anacardiaceae	*Loxopterygium huasango* Spruce ex Engl.
Apocynaceae	*Aspidosperma* sp.
	Aspidosperma sp.2
	Rauvolfia tetraphylla L.
	Vallesia glabra (Cav.) Link
	Prestonia mollis Kunth
Asteraceae	*Fulcaldea laurifolia* (Bonpl.) Poir.
	Spp. 1
Bignoniaceae	*Anemopaegma* sp.
	Handroanthus billbergii (Bureau & K.Schum.) S.O.Grose
	Handroanthus chrysanthus (Jacq.) S.O.Grose
	Tecoma stans (L.) Juss. ex Kunth
	Bignonia longiflora Cav.
Bixaceae	*Cochlospermum vitifolium* (Willd.) Spreng.
Boraginaceae	*Cordia alliodora* (Ruiz & Pav.) Oken
	Cordia lutea Lam.
	Cordia macrocephala (Desv.) Kunth
	Cordia sp.
Burseraceae	*Bursera graveolens* (Kunth) Triana & Planch.
Cannabaceae	*Celtis iguanaea* (Jacq.) Sarg.
	Celtis loxensis C.C. Berg
Capparaceae	*Colicodendron scabridum* (Kunth) Seem
	Cynophalla flexuosa (L.) J.Presl
	Cynophalla sclerophylla (Iltis & Cornejo) Cornejo & Iltis
Caricaceae	*Vasconcellea parviflora* A. DC.
Celastraceae	*Salacia* sp.
Combretaceae	*Terminalia valverdeae* A.H. Gentry
Convolvulaceae	*Ipomoea carnea* Jacq.
	Ipomoea wolcottiana subsp. calodendron (O'Donell) McPherson
Erythroxylaceae	*Erythroxylum glaucum* O. E. Schulz
Euphorbiaceae	*Croton* sp.
	Hura crepitans L.

Leguminosae	*Acacia macracantha* Humb. & Bonpl. Ex Willd.
	Albizia multiflora (Kunth) Barneby & J.W. Grimes
	Bauhinia aculeata L.
	Bauhinia sp.
	Caesalpinia spinosa (Molina) Kuntze
	Calliandra taxifolia (Kunth.) Benth.
	Chloroleucon mangense (Jack.) Britton & Rose
	Erythrina velutina Willd.
	Geoffroea spinosa Jacq.
	Leucaena trichodes (Jacq.) Benth.
	Machaerium millei Standl.
	Mimosa acantholoba (Willd.) Poir
	Piptadenia flava (Spreng. ex DC.) Benth.
	Piscidia carthagenensis Jacq.
	Pithecellobium excelsum (Kunth) Mart.
	Prosopis juliflora (Sw.) DC.
	Senna incarnata (Pav. & Benth.) H.S. Irwin & Barneby
	Centrolobium ochroxylum Rose ex Rudd
Malvaceae	*Ceiba insignis* (Kunth) P.E. Gibbs & Semir
	Eriotheca ruizii (K.Schum.) A. Robyns
	Guazuma ulmifolia Lam.
	Malvastrum sp.
	Byttneria sp.
	Waltheria ovata Cav.
Moraceae	*Maclura tinctoria* (L.) Steud.
Myrtaceae	*Psidium guajava* L.
Nyctaginaceae	*Bougainvillea peruviana* Bonpl.
	Pisonia aculeata L.
Opiliaceae	*Agonandra excelsa* Griseb.
Phytolaccaceae	*Gallesia integrifolia* (Spreng.) Harms
Piperaceae	*Piper* sp.
Polygonaceae	*Coccoloba ruiziana* Lindau
	Triplaris cumingiana Fisch. & C.A.Mey.
Rhamnaceae	*Ziziphus thyrsiflora* Benth.
Rubiaceae	*Randia armata* (Sw.) DC.
	Simira ecuadorensis (Standl.) Steyerm.
	Spp. 12
Salicaceae	*Prockia crucis* P. Browne ex L.
Sapindaceae	*Sapindus saponaria* L.
Solanaceae	*Acnistus arborescens* (L.) Schltdl
	Solanum albidum Dunal
Verbenaceae	*Citharexylum poeppigii* Walp
Unknown	Spp. 2
	Spp. 3
	Spp. 4
	Spp. 5
	Spp. 6
	Spp. 7
	Spp. 8
	Spp. 9
	Spp. 10
	Spp. 11
31	85

Supplementary Table S2. *P–values* that shows the correlation among variables. Values shaded on light grey means correlated variables.

	Goats	Cattle	Equine	HPI	Canp	SPrec	MTemp	Alt	Treat	OM	SDepth	Drain	Ston	Text
**Goats**	0													
**Cattle**	5.4×10^{-2}	0												
**Equine**	0.192	0.86	0											
**HPI.BA**	2.3×10^{-4}	4.9×10^{-3}	3.3×10^{-2}	0										
**Canp**	9.3×10^{-4}	5.9×10^{-2}	3.6×10^{-4}	1.2×10^{-27}	0									
**MPrec**	0.106	0.522	0.630	4.5×10^{-2}	0.780	0								
**MTemp**	3.4×10^{-12}	1.8×10^{-11}	7.9×10^{-8}	1.7×10^{-23}	1.6×10^{-14}	3.2×10^{-28}	0							
**Alt**	8.4×10^{-4}	1.1×10^{-2}	6.1×10^{-2}	2.0×10^{-7}	3.5×10^{-20}	6.7×10^{-2}	1.7×10^{-44}	0						
**Treat**	1.000	1.000	1.000	1.000	6.3×10^{-2}	0.696	0.699	1.000	0					
**OM**	2.0×10^{-3}	0.000	6.0×10^{-3}	0.000	1.0×10^{-3}	0.757	0.000	0.000	1.000	0				
**SDepth**	0.000	0.101	0.283	0.000	0.000	6.6×10^{-2}	0.000	0.000	1.000	0.000	0			
**Drain**	6.8×10^{-2}	1.0×10^{-2}	4.9×10^{-2}	0.000	0.000	0.259	0.000	1.5×10^{-2}	1.000	0.000	0.134	0		
**Ston**	0.000	0.738	0.875	0.169	1.3×10^{-2}	5.1×10^{-2}	0.000	0.000	1.000	0.000	7.0×10^{-3}	1.0×10^{-3}	0	
**Text**	0.938	4.5×10^{-2}	9.3×10^{-2}	0.000	0.000	0.110	0.000	2.2×10^{-2}	1.000	0.000	0.000	5.3×10^{-2}	2.6×10^{-2}	0

Supplementary Table S3. Candidate models assessed on richness, abundance and diversity

ID	Model	ID	Model	ID	Model
1	~1	41	~1+Goats+Treat+Drain+Text	81	~1+Cattle+Canp+SPrec+Treat
2	~1+Goats	42	~1+Goats+Cattle+Equine+SPrec+Treat	82	~1+Cattle+SPrec+Treat+SDepth
3	~1+Goats+Cattle	43	~1+Goats+Cattle+Equine+SPrec+Text	83	~1+Cattle+SPrec+Treat+Ston
4	~1+Goats+Equine	44	~1+Goats+Cattle+Equine+Treat+Text	84	~1+Cattle+SPrec+Treat+Text
5	~1+Goats+SPrec	45	~1+Goats+Cattle+Canp+SPrec+Treat	85	~1+Cattle+Equine+SPrec+Treat+SDepth
6	~1+Goats+Treat	46	~1+Goats+Cattle+SPrec+Treat+Text	86	~1+Cattle+Equine+SPrec+Treat+Ston
7	~1+Goats+Drain	47	~ 1+Goats+Equine+SPrec+Treat+Drain	87	~1+Cattle+Equine+SPrec+Treat+Text
8	~1+Goats+Text	48	~1+Goats+Equine+SPrec+Treat+Text	88	~1+Equine
9	~1+Goats+Cattle+Equine	49	~1+Goats+Equine+SPrec+Drain+Text	89	~1+Equine+SPrec
10	~1+Goats+Cattle+Canp	50	~1+Goats+Equine+Treat+Drain+Text	90	~1+Equine+Alt
11	~1+Goats+Cattle+SPrec	51	~1+Goats+SPrec+Treat+Drain+Text	91	~1+Equine+Treat
12	~1+Goats+Cattle+Treat	52	~1+Cattle	92	~1+Equine+SDepth
13	~1+Goats+Cattle+Text	53	~1+Cattle+Equine	93	~1+Equine+Drain
14	~1+Goats+Equine+SPrec	54	~1+Cattle+Canp	94	~1+Equine+Ston
15	~1+Goats+Equine+Treat	55	~1+Cattle+SPrec	95	~1+Equine+Text
16	~1+Goats+Equine+Drain	56	~1+Cattle+Treat	96	~1+Equine+SPrec+Alt
17	~1+Goats+Equine+Text	57	~1+Cattle+SDepth	97	~1+Equine+SPrec+Treat
18	~1+Goats+SPrec+Treat	58	~1+Cattle+Ston	98	~1+Equine+SPrec+SDepth
19	~1+Goats+SPrec+Drain	59	~1+Cattle+Text	99	~1+Equine+SPrec+Drain
20	~1+Goats+SPrec+Text	60	~1+Cattle+Equine+SPrec	100	~1+Equine+SPrec+Ston
21	~1+Goats+Treat+Drain	61	~1+Cattle+Equine+Treat	101	~1+Equine+SPrec+Text
22	~1+Goats+Treat+Text	62	~1+Cattle+Equine+SDepth	102	~1+Equine+Alt+Treat
23	~1+Goats+Drain+Text	63	~1+Cattle+Equine+Ston	103	~1+Equine+Treat+SDepth
24	~1+Goats+Cattle+Equine+SPrec	64	~1+Cattle+Equine+Text	104	~1+Equine+Treat+Drain
25	~1+Goats+Cattle+Equine+Treat	65	~1+Cattle+Canp+SPrec	105	~1+Equine+Treat+Ston
26	~1+Goats+Cattle+Equine+Text	66	~1+Cattle+Canp+Treat	106	~1+Equine+Treat+Text
27	~1+Goats+Cattle+Canp+SPrec	67	~1+Cattle+SPrec+Treat	107	~1+Equine+SDepth+Drain
28	~1+Goats+Cattle+Canp+Treat	68	~1+Cattle+SPrec+SDepth	108	~1+Equine+Drain+Text
29	~1+Goats+Cattle+SPrec+Treat	69	~1+Cattle+SPrec+Ston	109	~1+Equine+SPrec+Alt+Treat
30	~1+Goats+Cattle+SPrec+Text	70	~1+Cattle+SPrec+Text	110	~1+Equine+SPrec+Treat+SDepth
31	~1+Goats+Cattle+Treat+Text	71	~1+Cattle+Treat+SDepth	111	~1+Equine+SPrec+Treat+Drain
32	~1+Goats+Equine+SPrec+Treat	72	~1+Cattle+Treat+Ston	112	~1+Equine+SPrec+Treat+Ston
33	~1+Goats+Equine+SPrec+Drain	73	~1+Cattle+Treat+Text	113	~1+Equine+SPrec+Treat+Text
34	~1+Goats+Equine+SPrec+Text	74	~1+Cattle+Equine+SPrec+Treat	114	~1+Equine+SPrec+SDepth+Drain
35	~1+Goats+Equine+Treat+Drain	75	~1+Cattle+Equine+SPrec+SDepth	115	~1+Equine+SPrec+Drain+Text
36	~1+Goats+Equine+Treat+Text	76	~1+Cattle+Equine+SPrec+Ston	116	~1+Equine+Treat+SDepth+Drain
37	~1+Goats+Equine+Drain+Text	77	~1+Cattle+Equine+SPrec+Text	117	~1+Equine+Treat+Drain+Text
38	~1+Goats+SPrec+Treat+Drain	78	~1+Cattle+Equine+Treat+SDepth	118	~1+Equine+SPrec+Treat+SDepth+Drain
39	~1+Goats+SPrec+Treat+Text	79	~1+Cattle+Equine+Treat+Ston	119	~1+Equine+SPrec+Treat+Drain+Text
40	~1+Goats+SPrec+Drain+Text	80	~1+Cattle+Equine+Treat+Text	120	~1+HPI.BA

ID	Model	ID	Model
121	~1+HPI.BA+SPrec	154	~1+Treat
122	~1+HPI.BA+Treat	155	~1+Treat+OM
123	~1+HPI.BA+Ston	156	~1+Treat+SDepth
124	~1+HPI.BA+SPrec+Treat	157	~1+Treat+Drain
125	~1+HPI.BA+SPrec+Ston	158	~1+Treat+Ston
126	~1+HPI.BA+Treat+Ston	159	~1+Treat+Text
127	~1+HPI.BA+SPrec+Treat+Ston	160	~1+Treat+SDepth+Drain
128	~1+Canp	161	~1+Treat+Drain+Text
129	~1+Canp+SPrec	162	~1+OM
130	~1+Canp+Treat	163	~1+SDepth
131	~1+Canp+SPrec+Treat	164	~1+SDepth+Drain
132	~1+SPrec	165	~1+Drain
133	~1+SPrec+Alt	166	~1+Drain+Text
134	~1+SPrec+Treat	167	~1+Ston
135	~1+SPrec+OM	168	~1+Text
136	~1+SPrec+SDepth	169	~1+Goats*Cattle+SPrec+Treat
137	~1+SPrec+Drain	170	~1+Goats*Equine+SPrec+Treat
138	~1+SPrec+Ston	171	~1+Cattle*Equine+SPrec+Treat
139	~1+SPrec+Text	172	~1+Goats*Cattle*Equine+SPrec+Treat
140	~1+SPrec+Alt+Treat	173	~1+Goats*SPrec+Treat
141	~1+SPrec+Treat+OM	174	~1+Cattle*SPrec+Treat
142	~1+SPrec+Treat+SDepth	175	~1+Equine*SPrec+Treat
143	~1+SPrec+Treat+Drain	176	~1+HPI.BA*SPrec+Treat
144	~1+SPrec+Treat+Ston	177	~1+SPrec*Treat+OM
145	~1+SPrec+Treat+Text	178	~1+SPrec*OM+Treat
146	~1+SPrec+SDepth+Drain	179	~1+SPrec*SDepth+Treat
147	~1+SPrec+Drain+Text	180	~1+SPrec*Treat+SDepth
148	~1+SPrec+Treat+SDepth+Drain	181	~1+Goats*Treat+SPrec
149	~1+SPrec+Treat+Drain+Text	182	~1+Cattle*Treat+SPrec
150	~1+MTemp	183	~1+Equine*Treat+SPrec
151	~1+MTemp+Treat	184	~1+Equine*Treat+SPrec+Cattle
152	~1+Alt	185	~1+Equine*Treat+Cattle*Treat+SPrec
153	~1+Alt+Treat	186	~1+Goats*Cattle*Equine*Treat+SPrec

Supplementary Table S4. Ranges or levels of the predictors and transformation methods.

Predictor	Range or levels		Transformation method	Observations
	Min	Max		
Goats	0	1011	Standardised	Dry weight per transect in gr
Cattle	0	3225	Standardised	Dry weight per transect in gr
Equine	0	1282	Standardised	Dry weight per transect in gr
HPI	0	0.066	Log-transformed	Pressure index per plot. Basal area corresponds to mature forest. See Cueva *et al.*[26]
Canp	3.9	87.3		Percentage of canopy closure
SPrec	42	1005	Log-transformed	Precipitation in mm per plot
MTemp	22.9	26	Log-transformed	°C
Alt	211	890	Log-transformed	Meters above sea level
OM	Low<br>Medium<br>High			
SDepth	0-10<br>11-20<br>21-50<br>51-100<br>> 100			cm of soil depth
Drain	Good			Water is removed from the soil readily but not rapidly; water is available for plants*
	Moderate			Water is removed from the soil somewhat slowly during some periods of the year; the soils are wet for only a short time*
	Poor			Water is removed so slowly that the soil is wet at shallow depths*
Ston	Without<br>Very few<br>Few<br>Frequent<br>Abundant			
Text	Loam<br>Sandy loam<br>Clay loam<br>Clay–sandy loam			

* Description taken from: Soil Science Division Staff. Examination and Description of Soil Profiles. in *Soil Survey Manual* (eds. Ditzler, C., Scheffe, K. & Monger, H. C.) 83–234 (USDA Handbook 18, Government Printing Office, 2017).

Supplementary Table S5. Database to evaluate the structure and diversity of natural regeneration.

Measure	Time	Season	Cluster	Cod.plot	Treat	Ind	Spp	Simp	Goats	Cattle	Equine	HPI	Canp	SPrec	MTemp	Alt	OM	SDepth	Drain	Ston	Text
1	0	Dry	CL3	CL3P1	open	98	8	0.02522	0.20772	0.00000	0.00000	0.03442	0.51790	1.97772	1.38561	2.70728	Medium	21 - 50 cm	Good	Without	Loam
1	0	Dry	CL3	CL3P2	open	54	12	0.34940	0.36004	0.00000	0.00000	0.04057	0.51560	1.97772	1.38021	2.69955	Medium	21 - 50 cm	Good	Without	Loam
1	0	Dry	CL3	CL3P3	open	47	10	0.62395	0.32443	0.00000	0.00000	0.05310	0.48953	1.97772	1.38561	2.71912	Medium	21 - 50 cm	Good	Without	Loam
1	0	Dry	CL6	CL6P1	open	11	2	0.06615	0.23343	0.00000	0.00000	0.05110	0.13880	1.64345	1.39270	2.43057	Medium	0 - 10 cm	Good	Without	Sandy loam
1	0	Dry	CL6	CL6P2	open	0	0	0.00000	0.06924	0.00000	0.00000	0.06605	0.05618	1.63347	1.39620	2.40929	Medium	0 - 10 cm	Good	Without	Sandy loam
1	0	Dry	CL6	CL6P3	open	7	1	0.00000	0.12562	0.00000	0.00000	0.05876	0.06130	1.64345	1.39620	2.38998	Medium	0 - 10 cm	Good	Without	Sandy loam
1	0	Dry	CL7	CL7P1	open	20	2	0.00430	0.44115	0.00000	0.00000	0.05166	0.36963	1.65321	1.39794	2.32720	High	11 - 20 cm	Good	Abundant	Clay-sandy loam
1	0	Dry	CL7	CL7P2	open	15	3	0.24103	1.00000	0.00000	0.06396	0.04813	0.16270	1.65321	1.39794	2.33612	High	11 - 20 cm	Good	Abundant	Clay-sandy loam
1	0	Dry	CL7	CL7P3	open	11	3	0.20697	0.37784	0.00000	0.00000	0.03848	0.35183	1.66276	1.39620	2.35626	High	11 - 20 cm	Good	Abundant	Clay-sandy loam
1	0	Dry	CL8	CL8P1	open	48	7	0.44844	0.14045	0.00000	0.00000	0.02520	0.47975	1.84510	1.40483	2.60576	Medium	21 - 50 cm	Good	Without	Loam
1	0	Dry	CL8	CL8P2	open	87	7	0.28907	0.15430	0.20372	0.00780	0.03283	0.32945	1.84510	1.38561	2.57317	Medium	21 - 50 cm	Good	Without	Loam
1	0	Dry	CL8	CL8P3	open	36	6	0.15997	0.16024	0.00000	0.00000	0.02657	0.62873	1.86332	1.38561	2.58200	High	21 - 50 cm	Poorly drained	Few	Clay-sandy loam
1	0	Dry	CL11	CL11P1	open	27	6	0.18021	0.05836	0.01767	0.00000	0.01326	0.65428	1.88649	1.39794	2.54849	Medium	21 - 50 cm	Good	Without	Loam
1	0	Dry	CL11	CL11P2	open	132	10	0.24496	0.11078	0.00000	0.00000	0.01371	0.53023	1.89209	1.39794	2.55751	Medium	21 - 50 cm	Good	Without	Loam
1	0	Dry	CL11	CL11P3	open	7	6	0.85232	0.00000	0.00000	0.00000	0.01604	0.49678	1.89209	1.39794	2.54806	High	21 - 50 cm	Moderated	Without	Loam
1	0	Dry	CL12	CL12P1	open	68	9	0.12155	0.02374	0.00000	0.00000	0.02082	0.30320	2.33846	1.41497	2.46340	High	51 - 100 cm	Good	Frequent	Loam
1	0	Dry	CL12	CL12P2	open	15	6	0.46301	0.00000	0.00000	0.04056	0.02448	0.40963	2.34044	1.41497	2.41812	High	51 - 100 cm	Good	Frequent	Loam
1	0	Dry	CL12	CL12P3	open	16	3	0.17929	0.00000	0.34605	0.18643	0.02773	0.16573	2.33445	1.41497	2.38202	Low	11 - 20 cm	Moderated	Few	Clay loam
1	0	Dry	CL15	CL15P1	open	82	15	0.32173	0.38477	0.08744	0.00000	0.01088	0.48650	1.95904	1.42325	2.61065	High	> 100 cm	Moderated	Without	Loam
1	0	Dry	CL15	CL15P2	open	40	9	0.46406	0.12364	0.00000	0.00000	0.01328	0.46960	1.95904	1.42325	2.61507	High	21 - 50 cm	Moderated	Without	Loam
1	0	Dry	CL15	CL15P3	open	32	9	0.42516	0.26014	0.04620	0.00000	0.00991	0.60778	1.95904	1.42325	2.63075	High	> 100 cm	Moderated	Without	Loam
1	0	Dry	CL19	CL19P1	open	7	4	0.50056	0.00000	0.00000	0.00000	0.01080	0.35413	2.36549	1.38021	2.92670	Medium	51 - 100 cm	Good	Very few	Loam
1	0	Dry	CL19	CL19P2	open	15	5	0.22961	0.00000	0.21271	0.00000	0.01089	0.62780	2.36549	1.38021	2.91800	Medium	51 - 100 cm	Good	Very few	Loam
1	0	Dry	CL19	CL19P3	open	22	6	0.23269	0.00000	0.09767	0.00000	0.01667	0.60883	2.36736	1.38021	2.94979	Medium	51 - 100 cm	Good	Very few	Loam
1	0	Dry	CL21	CL21P1	open	21	3	0.14316	0.00000	0.00000	0.31123	0.00000	0.55740	1.94939	1.38382	2.85827	Low	51 - 100 cm	Moderated	Without	Clay-sandy loam
1	0	Dry	CL21	CL21P2	open	3	2	0.26503	0.00000	0.96620	0.00000	0.00000	0.58253	1.95424	1.38382	2.87564	Low	51 - 100 cm	Moderated	Without	Clay-sandy loam
1	0	Dry	CL21	CL21P3	open	8	4	0.52507	0.00000	0.32620	1.00000	0.00000	0.57465	1.94939	1.38382	2.85087	Low	51 - 100 cm	Moderated	Without	Clay-sandy loam
1	0	Dry	CL22	CL22P1	open	31	8	0.16858	0.00000	0.52899	0.18019	0.00000	0.62520	1.95424	1.38382	2.87564	Low	51 - 100 cm	Moderated	Without	Clay-sandy loam
1	0	Dry	CL22	CL22P2	open	31	7	0.38705	0.00000	0.07597	0.42902	0.00000	0.64065	1.94939	1.38382	2.85430	Low	51 - 100 cm	Moderated	Without	Clay-sandy loam
1	0	Dry	CL22	CL22P3	open	20	8	0.56961	0.00000	0.03504	0.18253	0.00000	0.63193	1.94448	1.38382	2.82674	Low	51 - 100 cm	Moderated	Without	Clay-sandy loam
1	0	Dry	CL23	CL23P1	open	2	2	1.00000	0.00000	0.19721	0.00000	0.00000	0.33094	1.87506	1.39094	2.81358	Low	51 - 100 cm	Moderated	Without	Clay-sandy loam
1	0	Dry	CL23	CL23P2	open	113	4	0.00144	0.00000	0.31442	0.12715	0.00000	0.40118	1.87506	1.39094	2.81381	Low	51 - 100 cm	Moderated	Without	Clay-sandy loam
1	0	Dry	CL23	CL23P3	open	41	4	0.08336	0.00000	0.40496	0.00000	0.00000	0.40533	1.87506	1.39094	2.81847	Low	51 - 100 cm	Moderated	Without	Clay-sandy loam
1	0	Dry	CL24	CL24P1	open	48	6	0.12107	0.00000	0.00000	0.98362	0.00000	0.53652	1.89209	1.38739	2.85126	Low	51 - 100 cm	Moderated	Without	Clay-sandy loam
1	0	Dry	CL24	CL24P2	open	29	9	0.42212	0.00000	0.30636	0.00000	0.00000	0.44705	1.89209	1.38739	2.84386	Low	51 - 100 cm	Moderated	Without	Clay-sandy loam
1	0	Dry	CL24	CL24P3	open	17	8	0.51415	0.00000	1.00000	0.00000	0.00000	0.26595	1.89209	1.38739	2.83059	Low	51 - 100 cm	Moderated	Without	Clay-sandy loam
2	7	Rainy	CL3	CL3P1	exclusion	60	13	0.51109	0.20772	0.00000	0.00000	0.03442	0.76583	2.78032	1.37840	2.70728	Medium	21 - 50 cm	Good	Without	Loam
2	7	Rainy	CL3	CL3P2	exclusion	109	12	0.54091	0.36004	0.00000	0.00000	0.04057	0.73233	2.78032	1.37840	2.69955	Medium	21 - 50 cm	Good	Without	Loam
2	7	Rainy	CL3	CL3P3	exclusion	102	15	0.45865	0.32443	0.00000	0.00000	0.05310	0.56828	2.78032	1.37840	2.71912	Medium	21 - 50 cm	Good	Without	Loam
2	7	Rainy	CL6	CL6P1	exclusion	15	1	0.00000	0.23343	0.00000	0.00000	0.05110	0.09773	2.64444	1.38382	2.43057	Medium	0 - 10 cm	Good	Without	Sandy loam
2	7	Rainy	CL6	CL6P2	exclusion	8	3	0.19511	0.06924	0.00000	0.00000	0.06605	0.25003	2.61909	1.38917	2.40929	Medium	0 - 10 cm	Good	Without	Sandy loam
2	7	Rainy	CL6	CL6P3	exclusion	11	1	0.00000	0.12562	0.00000	0.00000	0.05876	0.08515	2.62634	1.38917	2.38998	Medium	0 - 10 cm	Good	Without	Sandy loam
2	7	Rainy	CL7	CL7P1	exclusion	91	4	0.01522	0.44115	0.00000	0.00000	0.05166	0.61218	2.60097	1.38917	2.32720	High	11 - 20 cm	Good	Abundant	Clay-sandy loam
2	7	Rainy	CL7	CL7P2	exclusion	46	3	0.11240	1.00000	0.00000	0.06396	0.04813	0.68053	2.60097	1.38917	2.33612	High	11 - 20 cm	Good	Abundant	Clay-sandy loam
2	7	Rainy	CL7	CL7P3	exclusion	77	7	0.38544	0.37784	0.00000	0.00000	0.03848	0.63038	2.61490	1.38917	2.35626	High	11 - 20 cm	Good	Abundant	Clay-sandy loam
2	7	Rainy	CL8	CL8P1	exclusion	60	6	0.33026	0.14045	0.00000	0.00000	0.02520	0.62758	2.67025	1.37291	2.60576	Medium	21 - 50 cm	Good	Without	Loam
2	7	Rainy	CL8	CL8P2	exclusion	39	7	0.34835	0.15430	0.20372	0.00780	0.03283	0.67353	2.66087	1.37475	2.57317	Medium	21 - 50 cm	Good	Without	Loam
2	7	Rainy	CL8	CL8P3	exclusion	27	5	0.20703	0.16024	0.00000	0.00000	0.02657	0.71990	2.66839	1.37475	2.58200	High	21 - 50 cm	Poorly drained	Few	Clay-sandy loam
2	7	Rainy	CL11	CL11P1	exclusion	87	11	0.13139	0.05836	0.01767	0.00000	0.01326	0.78655	2.76938	1.38917	2.54849	Medium	21 - 50 cm	Good	Without	Loam
2	7	Rainy	CL11	CL11P2	exclusion	62	12	0.52471	0.11078	0.00000	0.00000	0.01371	0.75580	2.76492	1.38917	2.55751	Medium	21 - 50 cm	Good	Without	Loam
2	7	Rainy	CL11	CL11P3	exclusion	92	14	0.56641	0.00000	0.00000	0.00000	0.01604	0.80893	2.76492	1.38917	2.54806	High	21 - 50 cm	Moderated	Without	Loam

Measure	Time	Season	Cluster	Cod.plot	Treat	Ind	Spp	Simp	Goats	Cattle	Equine	HPI	Canp	SPrec	MTemp	Alt	OM	SDepth	Drain	Ston	Text
2	7	Rainy	CL12	CL12P1	exclusion	146	10	0.08232	0.02374	0.00000	0.00000	0.02082	0.71603	2.74429	1.38021	2.46340	High	51 - 100 cm	Good	Frequent	Loam
2	7	Rainy	CL12	CL12P2	exclusion	78	11	0.44362	0.00000	0.00000	0.04056	0.02448	0.70158	2.74429	1.38021	2.41812	High	51 - 100 cm	Good	Frequent	Loam
2	7	Rainy	CL12	CL12P3	exclusion	139	16	0.57618	0.00000	0.34605	0.18643	0.02773	0.58263	2.74429	1.38021	2.38202	Low	11 - 20 cm	Moderated	Few	Clay loam
2	7	Rainy	CL15	CL15P1	exclusion	72	12	0.31255	0.38477	0.08744	0.00000	0.01088	0.65075	2.78746	1.38202	2.61065	High	> 100 cm	Moderated	Without	Loam
2	7	Rainy	CL15	CL15P2	exclusion	55	10	0.46578	0.12364	0.00000	0.00000	0.01328	0.70950	2.78746	1.38382	2.61507	High	21 - 50 cm	Moderated	Without	Loam
2	7	Rainy	CL15	CL15P3	exclusion	70	15	0.31246	0.26014	0.04620	0.00000	0.00991	0.80575	2.78746	1.38202	2.63075	High	> 100 cm	Moderated	Without	Loam
2	7	Rainy	CL19	CL19P1	exclusion	28	4	0.20792	0.00000	0.00000	0.00000	0.01080	0.61168	3.00260	1.36736	2.92670	Medium	51 - 100 cm	Good	Very few	Loam
2	7	Rainy	CL19	CL19P2	exclusion	17	5	0.33063	0.00000	0.21271	0.00000	0.01089	0.74540	3.00260	1.36736	2.91800	Medium	51 - 100 cm	Good	Very few	Loam
2	7	Rainy	CL19	CL19P3	exclusion	56	12	0.43696	0.00000	0.09767	0.00000	0.01667	0.75230	3.00260	1.36736	2.94979	Medium	51 - 100 cm	Good	Very few	Loam
2	7	Rainy	CL21	CL21P1	exclusion	61	10	0.27440	0.00000	0.00000	0.31123	0.00000	0.75283	2.73719	1.35025	2.85827	Low	51 - 100 cm	Moderated	Without	Clay-sandy loam
2	7	Rainy	CL21	CL21P2	exclusion	26	6	0.22824	0.00000	0.96620	0.00000	0.00000	0.70473	2.73719	1.35025	2.87564	Low	51 - 100 cm	Moderated	Without	Clay-sandy loam
2	7	Rainy	CL21	CL21P3	exclusion	53	5	0.17197	0.00000	0.32620	1.00000	0.00000	0.76800	2.73719	1.35025	2.85087	Low	51 - 100 cm	Moderated	Without	Clay-sandy loam
2	7	Rainy	CL22	CL22P1	exclusion	63	9	0.50785	0.00000	0.52899	0.18019	0.00000	0.79838	2.73719	1.35025	2.87564	Low	51 - 100 cm	Moderated	Without	Clay-sandy loam
2	7	Rainy	CL22	CL22P2	exclusion	35	16	0.73228	0.00000	0.07597	0.42902	0.00000	0.71855	2.73719	1.35218	2.85430	Low	51 - 100 cm	Moderated	Without	Clay-sandy loam
2	7	Rainy	CL22	CL22P3	exclusion	60	8	0.33284	0.00000	0.03504	0.18253	0.00000	0.75030	2.73719	1.35218	2.82674	Low	51 - 100 cm	Moderated	Without	Clay-sandy loam
2	7	Rainy	CL23	CL23P1	exclusion	26	4	0.26637	0.00000	0.19721	0.00000	0.00000	0.60893	2.68485	1.35025	2.81358	Low	51 - 100 cm	Moderated	Without	Clay-sandy loam
2	7	Rainy	CL23	CL23P2	exclusion	97	11	0.44695	0.00000	0.31442	0.12715	0.00000	0.59985	2.68485	1.35025	2.81381	Low	51 - 100 cm	Moderated	Without	Clay-sandy loam
2	7	Rainy	CL23	CL23P3	exclusion	163	7	0.46451	0.00000	0.40496	0.00000	0.00000	0.70660	2.68485	1.35025	2.81847	Low	51 - 100 cm	Moderated	Without	Clay-sandy loam
2	7	Rainy	CL24	CL24P1	exclusion	448	5	0.00618	0.00000	0.00000	0.98362	0.00000	0.75795	2.70243	1.34635	2.85126	Low	51 - 100 cm	Moderated	Without	Clay-sandy loam
2	7	Rainy	CL24	CL24P2	exclusion	331	7	0.20677	0.00000	0.30636	0.00000	0.00000	0.74030	2.70243	1.34635	2.84386	Low	51 - 100 cm	Moderated	Without	Clay-sandy loam
2	7	Rainy	CL24	CL24P3	exclusion	77	6	0.04656	0.00000	1.00000	0.00000	0.00000	0.75603	2.69897	1.34830	2.83059	Low	51 - 100 cm	Moderated	Without	Clay-sandy loam
2	7	Rainy	CL3	CL3P1	open	139	16	0.24112	0.20772	0.00000	0.00000	0.03442	0.71515	2.78032	1.37840	2.70728	Medium	21 - 50 cm	Good	Without	Loam
2	7	Rainy	CL3	CL3P2	open	62	15	0.69612	0.36004	0.00000	0.00000	0.04057	0.81495	2.78032	1.37840	2.69955	Medium	21 - 50 cm	Good	Without	Loam
2	7	Rainy	CL3	CL3P3	open	138	17	0.35126	0.32443	0.00000	0.00000	0.05310	0.62510	2.78032	1.37840	2.71912	Medium	21 - 50 cm	Good	Without	Loam
2	7	Rainy	CL6	CL6P1	open	17	2	0.00090	0.23343	0.00000	0.00000	0.05110	0.15363	2.64444	1.38382	2.43057	Medium	0 - 10 cm	Good	Without	Sandy loam
2	7	Rainy	CL6	CL6P2	open	105	1	0.00000	0.06924	0.00000	0.00000	0.06605	0.06750	2.61909	1.38917	2.40929	Medium	0 - 10 cm	Good	Without	Sandy loam
2	7	Rainy	CL6	CL6P3	open	28	2	0.00018	0.12562	0.00000	0.00000	0.05876	0.11545	2.62634	1.38917	2.38998	Medium	0 - 10 cm	Good	Without	Sandy loam
2	7	Rainy	CL7	CL7P1	open	23	3	0.05971	0.44115	0.00000	0.00000	0.05166	0.56403	2.60097	1.38917	2.32720	High	11 - 20 cm	Good	Abundant	Clay-sandy loam
2	7	Rainy	CL7	CL7P2	open	113	4	0.00208	1.00000	0.00000	0.06396	0.04813	0.33560	2.60097	1.38917	2.33612	High	11 - 20 cm	Good	Abundant	Clay-sandy loam
2	7	Rainy	CL7	CL7P3	open	54	6	0.18942	0.37784	0.00000	0.00000	0.03848	0.68780	2.61490	1.38917	2.35626	High	11 - 20 cm	Good	Abundant	Clay-sandy loam
2	7	Rainy	CL8	CL8P1	open	80	9	0.41175	0.14045	0.00000	0.00000	0.02520	0.61515	2.67025	1.37291	2.60576	Medium	21 - 50 cm	Good	Without	Loam
2	7	Rainy	CL8	CL8P2	open	113	9	0.41088	0.15430	0.20372	0.00780	0.03283	0.46080	2.66087	1.37475	2.57317	Medium	21 - 50 cm	Good	Without	Loam
2	7	Rainy	CL8	CL8P3	open	29	3	0.14682	0.16024	0.00000	0.00000	0.02657	0.67270	2.66839	1.37475	2.58200	High	21 - 50 cm	Poorly drained	Few	Clay-sandy loam
2	7	Rainy	CL11	CL11P1	open	63	12	0.45442	0.05836	0.01767	0.00000	0.01326	0.80360	2.76938	1.38917	2.54849	Medium	21 - 50 cm	Good	Without	Loam
2	7	Rainy	CL11	CL11P2	open	225	14	0.40665	0.11078	0.00000	0.00000	0.01371	0.78225	2.76492	1.38917	2.55751	Medium	21 - 50 cm	Good	Without	Loam
2	7	Rainy	CL11	CL11P3	open	28	12	0.62210	0.00000	0.00000	0.00000	0.01604	0.70743	2.76492	1.38917	2.54806	High	21 - 50 cm	Moderated	Without	Loam
2	7	Rainy	CL12	CL12P1	open	72	9	0.19353	0.02374	0.00000	0.00000	0.02082	0.73198	2.74429	1.38021	2.46340	High	51 - 100 cm	Good	Frequent	Loam
2	7	Rainy	CL12	CL12P2	open	42	9	0.49587	0.00000	0.00000	0.04056	0.02448	0.61255	2.74429	1.38021	2.41812	High	51 - 100 cm	Good	Frequent	Loam
2	7	Rainy	CL12	CL12P3	open	117	11	0.38716	0.00000	0.34605	0.18643	0.02773	0.37900	2.74429	1.38021	2.38202	Low	11 - 20 cm	Moderated	Few	Clay loam
2	7	Rainy	CL15	CL15P1	open	103	11	0.28512	0.38477	0.08744	0.00000	0.01088	0.77295	2.78746	1.38202	2.61065	High	> 100 cm	Moderated	Without	Loam
2	7	Rainy	CL15	CL15P2	open	66	9	0.35242	0.12364	0.00000	0.00000	0.01328	0.71863	2.78746	1.38382	2.61507	High	21 - 50 cm	Moderated	Without	Loam
2	7	Rainy	CL15	CL15P3	open	65	14	0.64229	0.26014	0.04620	0.00000	0.00991	0.87275	2.78746	1.38202	2.63075	High	> 100 cm	Moderated	Without	Loam
2	7	Rainy	CL19	CL19P1	open	22	7	0.57416	0.00000	0.00000	0.00000	0.01080	0.58868	3.00260	1.36736	2.92670	Medium	51 - 100 cm	Good	Very few	Loam
2	7	Rainy	CL19	CL19P2	open	29	5	0.03835	0.00000	0.21271	0.00000	0.01089	0.65015	3.00260	1.36736	2.91800	Medium	51 - 100 cm	Good	Very few	Loam
2	7	Rainy	CL19	CL19P3	open	40	6	0.21816	0.00000	0.09767	0.00000	0.01667	0.64088	3.00260	1.36736	2.94979	Medium	51 - 100 cm	Good	Very few	Loam
2	7	Rainy	CL21	CL21P1	open	69	11	0.46018	0.00000	0.00000	0.31123	0.00000	0.78733	2.73719	1.35025	2.85827	Low	51 - 100 cm	Moderated	Without	Clay-sandy loam
2	7	Rainy	CL21	CL21P2	open	34	4	0.09007	0.00000	0.96620	0.00000	0.00000	0.69090	2.73719	1.35025	2.87564	Low	51 - 100 cm	Moderated	Without	Clay-sandy loam
2	7	Rainy	CL21	CL21P3	open	65	12	0.43397	0.00000	0.32620	1.00000	0.00000	0.76613	2.73719	1.35025	2.85087	Low	51 - 100 cm	Moderated	Without	Clay-sandy loam
2	7	Rainy	CL22	CL22P1	open	57	9	0.49173	0.00000	0.52899	0.18019	0.00000	0.77420	2.73719	1.35025	2.87564	Low	51 - 100 cm	Moderated	Without	Clay-sandy loam
2	7	Rainy	CL22	CL22P2	open	50	10	0.37370	0.00000	0.07597	0.42902	0.00000	0.72450	2.73719	1.35218	2.85430	Low	51 - 100 cm	Moderated	Without	Clay-sandy loam
2	7	Rainy	CL22	CL22P3	open	40	8	0.52853	0.00000	0.03504	0.18253	0.00000	0.76255	2.73719	1.35218	2.82674	Low	51 - 100 cm	Moderated	Without	Clay-sandy loam
2	7	Rainy	CL23	CL23P1	open	33	6	0.23886	0.00000	0.19721	0.00000	0.00000	0.67263	2.68485	1.35025	2.81358	Low	51 - 100 cm	Moderated	Without	Clay-sandy loam
2	7	Rainy	CL23	CL23P2	open	518	11	0.08226	0.00000	0.31442	0.12715	0.00000	0.67373	2.68485	1.35025	2.81381	Low	51 - 100 cm	Moderated	Without	Clay-sandy loam

Measure	Time	Season	Cluster	Cod.plot	Treat	Ind	Spp	Simp	Goats	Cattle	Equine	HPI	Canp	SPrec	MTemp	Alt	OM	SDepth	Drain	Ston	Text
2	7	Rainy	CL23	CL23P3	open	178	10	0.53699	0.00000	0.40496	0.00000	0.00000	0.69535	2.68485	1.35025	2.81847	Low	51 - 100 cm	Moderated	Without	Clay-sandy loam
2	7	Rainy	CL24	CL24P1	open	94	10	0.39911	0.00000	0.00000	0.98362	0.00000	0.76983	2.70243	1.34635	2.85126	Low	51 - 100 cm	Moderated	Without	Clay-sandy loam
2	7	Rainy	CL24	CL24P2	open	266	11	0.12259	0.00000	0.30636	0.00000	0.00000	0.68478	2.70243	1.34635	2.84386	Low	51 - 100 cm	Moderated	Without	Clay-sandy loam
2	7	Rainy	CL24	CL24P3	open	116	9	0.41044	0.00000	1.00000	0.00000	0.00000	0.65275	2.69897	1.34830	2.83059	Low	51 - 100 cm	Moderated	Without	Clay-sandy loam
3	14	Dry	CL3	CL3P1	exclusion	61	15	0.57928	0.20772	0.00000	0.00000	0.03442	0.58740	1.97772	1.41330	2.70728	Medium	21 - 50 cm	Good	Without	Loam
3	14	Dry	CL3	CL3P2	exclusion	121	21	0.64494	0.36004	0.00000	0.00000	0.04057	0.55635	1.97772	1.41330	2.69955	Medium	21 - 50 cm	Good	Without	Loam
3	14	Dry	CL3	CL3P3	exclusion	70	14	0.60360	0.32443	0.00000	0.00000	0.05310	0.34878	1.97772	1.41330	2.71912	Medium	21 - 50 cm	Good	Without	Loam
3	14	Dry	CL6	CL6P1	exclusion	13	1	0.00000	0.23343	0.00000	0.00000	0.05110	0.03913	1.64345	1.43297	2.43057	Medium	0 - 10 cm	Good	Without	Sandy loam
3	14	Dry	CL6	CL6P2	exclusion	4	1	0.00000	0.06924	0.00000	0.00000	0.06605	0.18193	1.63347	1.43933	2.40929	Medium	0 - 10 cm	Good	Without	Sandy loam
3	14	Dry	CL6	CL6P3	exclusion	7	1	0.00000	0.12562	0.00000	0.00000	0.05876	0.05578	1.64345	1.43933	2.38998	Medium	0 - 10 cm	Good	Without	Sandy loam
3	14	Dry	CL7	CL7P1	exclusion	67	4	0.00305	0.44115	0.00000	0.00000	0.05166	0.37645	1.65321	1.44091	2.32720	High	11 - 20 cm	Good	Abundant	Clay-sandy loam
3	14	Dry	CL7	CL7P2	exclusion	21	4	0.08520	1.00000	0.00000	0.06396	0.04813	0.29590	1.65321	1.44091	2.33612	High	11 - 20 cm	Good	Abundant	Clay-sandy loam
3	14	Dry	CL7	CL7P3	exclusion	48	5	0.26388	0.37784	0.00000	0.00000	0.03848	0.39880	1.66276	1.43933	2.35626	High	11 - 20 cm	Good	Abundant	Clay-sandy loam
3	14	Dry	CL8	CL8P1	exclusion	108	8	0.40426	0.14045	0.00000	0.00000	0.02520	0.60438	1.84510	1.41664	2.60576	Medium	21 - 50 cm	Good	Without	Loam
3	14	Dry	CL8	CL8P2	exclusion	66	7	0.26747	0.15430	0.20372	0.00780	0.03283	0.55603	1.84510	1.41996	2.57317	Medium	21 - 50 cm	Good	Without	Loam
3	14	Dry	CL8	CL8P3	exclusion	108	10	0.26797	0.16024	0.00000	0.00000	0.02657	0.46150	1.86332	1.42160	2.58200	High	21 - 50 cm	Poorly drained	Few	Clay-sandy loam
3	14	Dry	CL11	CL11P1	exclusion	83	11	0.12580	0.05836	0.01767	0.00000	0.01326	0.61230	1.88649	1.42488	2.54849	Medium	21 - 50 cm	Good	Without	Loam
3	14	Dry	CL11	CL11P2	exclusion	42	9	0.47970	0.11078	0.00000	0.00000	0.01371	0.54073	1.89209	1.42488	2.55751	Medium	21 - 50 cm	Good	Without	Loam
3	14	Dry	CL11	CL11P3	exclusion	81	14	0.56387	0.00000	0.00000	0.00000	0.01604	0.40843	1.89209	1.42488	2.54806	High	21 - 50 cm	Moderated	Without	Loam
3	14	Dry	CL12	CL12P1	exclusion	67	12	0.39501	0.02374	0.00000	0.00000	0.02082	0.26463	2.33846	1.43136	2.46340	High	51 - 100 cm	Good	Frequent	Loam
3	14	Dry	CL12	CL12P2	exclusion	127	12	0.11909	0.00000	0.00000	0.04056	0.02448	0.29288	2.34044	1.43136	2.41812	High	51 - 100 cm	Good	Frequent	Loam
3	14	Dry	CL12	CL12P3	exclusion	94	15	0.55661	0.00000	0.34605	0.18643	0.02773	0.23843	2.33445	1.43136	2.38202	Low	11 - 20 cm	Moderated	Few	Clay loam
3	14	Dry	CL15	CL15P1	exclusion	50	12	0.50775	0.38477	0.08744	0.00000	0.01088	0.52773	1.95904	1.41996	2.61065	High	> 100 cm	Moderated	Without	Loam
3	14	Dry	CL15	CL15P2	exclusion	50	10	0.46562	0.12364	0.00000	0.00000	0.01328	0.45038	1.95904	1.41996	2.61507	High	21 - 50 cm	Moderated	Without	Loam
3	14	Dry	CL15	CL15P3	exclusion	73	15	0.34328	0.26014	0.04620	0.00000	0.00991	0.58673	1.95904	1.41996	2.63075	High	> 100 cm	Moderated	Without	Loam
3	14	Dry	CL19	CL19P1	exclusion	24	4	0.19294	0.00000	0.00000	0.00000	0.01080	0.44185	2.36549	1.37840	2.92670	Medium	51 - 100 cm	Good	Very few	Loam
3	14	Dry	CL19	CL19P2	exclusion	18	5	0.38150	0.00000	0.21271	0.00000	0.01089	0.58910	2.36549	1.37840	2.91800	Medium	51 - 100 cm	Good	Very few	Loam
3	14	Dry	CL19	CL19P3	exclusion	58	13	0.44508	0.00000	0.09767	0.00000	0.01667	0.65665	2.36736	1.37840	2.94979	Medium	51 - 100 cm	Good	Very few	Loam
3	14	Dry	CL21	CL21P1	exclusion	63	11	0.34969	0.00000	0.00000	0.31123	0.00000	0.59710	1.94939	1.39094	2.85827	Low	51 - 100 cm	Moderated	Without	Clay-sandy loam
3	14	Dry	CL21	CL21P2	exclusion	25	9	0.33586	0.00000	0.96620	0.00000	0.00000	0.65285	1.95424	1.39094	2.87564	Low	51 - 100 cm	Moderated	Without	Clay-sandy loam
3	14	Dry	CL21	CL21P3	exclusion	42	4	0.06925	0.00000	0.32620	1.00000	0.00000	0.57120	1.94939	1.39094	2.85087	Low	51 - 100 cm	Moderated	Without	Clay-sandy loam
3	14	Dry	CL22	CL22P1	exclusion	51	9	0.50511	0.00000	0.52899	0.18019	0.00000	0.72773	1.95424	1.39094	2.87564	Low	51 - 100 cm	Moderated	Without	Clay-sandy loam
3	14	Dry	CL22	CL22P2	exclusion	33	14	0.73688	0.00000	0.07597	0.42902	0.00000	0.65948	1.94939	1.39094	2.85430	Low	51 - 100 cm	Moderated	Without	Clay-sandy loam
3	14	Dry	CL22	CL22P3	exclusion	42	7	0.38830	0.00000	0.03504	0.18253	0.00000	0.65170	1.94448	1.39094	2.82674	Low	51 - 100 cm	Moderated	Without	Clay-sandy loam
3	14	Dry	CL23	CL23P1	exclusion	34	4	0.17942	0.00000	0.19721	0.00000	0.00000	0.27673	1.87506	1.39445	2.81358	Low	51 - 100 cm	Moderated	Without	Clay-sandy loam
3	14	Dry	CL23	CL23P2	exclusion	81	10	0.36363	0.00000	0.31442	0.12715	0.00000	0.30560	1.87506	1.39445	2.81381	Low	51 - 100 cm	Moderated	Without	Clay-sandy loam
3	14	Dry	CL23	CL23P3	exclusion	110	7	0.46272	0.00000	0.40496	0.00000	0.00000	0.40588	1.87506	1.39445	2.81847	Low	51 - 100 cm	Moderated	Without	Clay-sandy loam
3	14	Dry	CL24	CL24P1	exclusion	412	5	0.00009	0.00000	0.00000	0.98362	0.00000	0.57357	1.89209	1.39094	2.85126	Low	51 - 100 cm	Moderated	Without	Clay-sandy loam
3	14	Dry	CL24	CL24P2	exclusion	156	8	0.41784	0.00000	0.30636	0.00000	0.00000	0.47620	1.89209	1.39094	2.84386	Low	51 - 100 cm	Moderated	Without	Clay-sandy loam
3	14	Dry	CL24	CL24P3	exclusion	63	6	0.01279	0.00000	1.00000	0.00000	0.00000	0.47125	1.89209	1.39094	2.83059	Low	51 - 100 cm	Moderated	Without	Clay-sandy loam
3	14	Dry	CL3	CL3P1	open	130	17	0.24274	0.20772	0.00000	0.00000	0.03442	0.51790	1.97772	1.41330	2.70728	Medium	21 - 50 cm	Good	Without	Loam
3	14	Dry	CL3	CL3P2	open	64	16	0.71408	0.36004	0.00000	0.00000	0.04057	0.51560	1.97772	1.41330	2.69955	Medium	21 - 50 cm	Good	Without	Loam
3	14	Dry	CL3	CL3P3	open	125	16	0.43700	0.32443	0.00000	0.00000	0.05310	0.48953	1.97772	1.41330	2.71912	Medium	21 - 50 cm	Good	Without	Loam
3	14	Dry	CL6	CL6P1	open	21	3	0.00403	0.23343	0.00000	0.00000	0.05110	0.13880	1.64345	1.43297	2.43057	Medium	0 - 10 cm	Good	Without	Sandy loam
3	14	Dry	CL6	CL6P2	open	50	1	0.00000	0.06924	0.00000	0.00000	0.06605	0.05618	1.63347	1.43933	2.40929	Medium	0 - 10 cm	Good	Without	Sandy loam
3	14	Dry	CL6	CL6P3	open	14	2	0.00171	0.12562	0.00000	0.00000	0.05876	0.06130	1.64345	1.43933	2.38998	Medium	0 - 10 cm	Good	Without	Sandy loam
3	14	Dry	CL7	CL7P1	open	16	2	0.02520	0.44115	0.00000	0.00000	0.05166	0.36963	1.65321	1.44091	2.32720	High	11 - 20 cm	Good	Abundant	Clay-sandy loam
3	14	Dry	CL7	CL7P2	open	51	4	0.00706	1.00000	0.00000	0.06396	0.04813	0.16270	1.65321	1.44091	2.33612	High	11 - 20 cm	Good	Abundant	Clay-sandy loam
3	14	Dry	CL7	CL7P3	open	17	4	0.20638	0.37784	0.00000	0.00000	0.03848	0.35183	1.66276	1.43933	2.35626	High	11 - 20 cm	Good	Abundant	Clay-sandy loam
3	14	Dry	CL8	CL8P1	open	110	11	0.30721	0.14045	0.00000	0.00000	0.02520	0.47975	1.84510	1.41664	2.60576	Medium	21 - 50 cm	Good	Without	Loam
3	14	Dry	CL8	CL8P2	open	135	10	0.40897	0.15430	0.20372	0.00780	0.03283	0.32945	1.84510	1.41996	2.57317	Medium	21 - 50 cm	Good	Without	Loam
3	14	Dry	CL8	CL8P3	open	41	5	0.10749	0.16024	0.00000	0.00000	0.02657	0.62873	1.86332	1.42160	2.58200	High	21 - 50 cm	Poorly drained	Few	Clay-sandy loam
3	14	Dry	CL11	CL11P1	open	54	12	0.43909	0.05836	0.01767	0.00000	0.01326	0.65428	1.88649	1.42488	2.54849	Medium	21 - 50 cm	Good	Without	Loam

Measure	Time	Season	Cluster	Cod.plot	Treat	Ind	Spp	Simp	Goats	Cattle	Equine	HPI	Canp	SPrec	MTemp	Alt	OM	SDepth	Drain	Ston	Text
3	14	Dry	CL11	CL11P2	open	175	11	0.35485	0.11078	0.00000	0.00000	0.01371	0.53023	1.89209	1.42488	2.55751	Medium	21 - 50 cm	Good	Without	Loam
3	14	Dry	CL11	CL11P3	open	17	10	0.70063	0.00000	0.00000	0.00000	0.01604	0.49678	1.89209	1.42488	2.54806	High	21 - 50 cm	Moderated	Without	Loam
3	14	Dry	CL12	CL12P1	open	69	9	0.14649	0.02374	0.00000	0.00000	0.02082	0.30320	2.33846	1.43136	2.46340	High	51 - 100 cm	Good	Frequent	Loam
3	14	Dry	CL12	CL12P2	open	29	8	0.49229	0.00000	0.00000	0.04056	0.02448	0.40963	2.34044	1.43136	2.41812	High	51 - 100 cm	Good	Frequent	Loam
3	14	Dry	CL12	CL12P3	open	52	8	0.50603	0.00000	0.34605	0.18643	0.02773	0.16573	2.33445	1.43136	2.38202	Low	11 - 20 cm	Moderated	Few	Clay loam
3	14	Dry	CL15	CL15P1	open	99	11	0.26236	0.38477	0.08744	0.00000	0.01088	0.48650	1.95904	1.41996	2.61065	High	> 100 cm	Moderated	Without	Loam
3	14	Dry	CL15	CL15P2	open	74	13	0.44061	0.12364	0.00000	0.00000	0.01328	0.46960	1.95904	1.41996	2.61507	High	21 - 50 cm	Moderated	Without	Loam
3	14	Dry	CL15	CL15P3	open	59	14	0.65268	0.26014	0.04620	0.00000	0.00991	0.60778	1.95904	1.41996	2.63075	High	> 100 cm	Moderated	Without	Loam
3	14	Dry	CL19	CL19P1	open	21	7	0.59269	0.00000	0.00000	0.00000	0.01080	0.35413	2.36549	1.37840	2.92670	Medium	51 - 100 cm	Good	Very few	Loam
3	14	Dry	CL19	CL19P2	open	24	5	0.06536	0.00000	0.21271	0.00000	0.01089	0.62780	2.36549	1.37840	2.91800	Medium	51 - 100 cm	Good	Very few	Loam
3	14	Dry	CL19	CL19P3	open	36	6	0.24492	0.00000	0.09767	0.00000	0.01667	0.60883	2.36736	1.37840	2.94979	Medium	51 - 100 cm	Good	Very few	Loam
3	14	Dry	CL21	CL21P1	open	54	11	0.52439	0.00000	0.00000	0.31123	0.00000	0.55740	1.94939	1.39094	2.85827	Low	51 - 100 cm	Moderated	Without	Clay-sandy loam
3	14	Dry	CL21	CL21P2	open	9	4	0.38979	0.00000	0.96620	0.00000	0.00000	0.58253	1.95424	1.39094	2.87564	Low	51 - 100 cm	Moderated	Without	Clay-sandy loam
3	14	Dry	CL21	CL21P3	open	40	11	0.62282	0.00000	0.32620	1.00000	0.00000	0.57465	1.94939	1.39094	2.85087	Low	51 - 100 cm	Moderated	Without	Clay-sandy loam
3	14	Dry	CL22	CL22P1	open	48	9	0.39738	0.00000	0.52899	0.18019	0.00000	0.62520	1.95424	1.39094	2.87564	Low	51 - 100 cm	Moderated	Without	Clay-sandy loam
3	14	Dry	CL22	CL22P2	open	42	10	0.34296	0.00000	0.07597	0.42902	0.00000	0.64065	1.94939	1.39094	2.85430	Low	51 - 100 cm	Moderated	Without	Clay-sandy loam
3	14	Dry	CL22	CL22P3	open	27	8	0.51135	0.00000	0.03504	0.18253	0.00000	0.63193	1.94448	1.39094	2.82674	Low	51 - 100 cm	Moderated	Without	Clay-sandy loam
3	14	Dry	CL23	CL23P1	open	35	7	0.29534	0.00000	0.19721	0.00000	0.00000	0.33094	1.87506	1.39445	2.81358	Low	51 - 100 cm	Moderated	Without	Clay-sandy loam
3	14	Dry	CL23	CL23P2	open	128	11	0.19050	0.00000	0.31442	0.12715	0.00000	0.40118	1.87506	1.39445	2.81381	Low	51 - 100 cm	Moderated	Without	Clay-sandy loam
3	14	Dry	CL23	CL23P3	open	118	11	0.48485	0.00000	0.40496	0.00000	0.00000	0.40533	1.87506	1.39445	2.81847	Low	51 - 100 cm	Moderated	Without	Clay-sandy loam
3	14	Dry	CL24	CL24P1	open	57	6	0.12654	0.00000	0.00000	0.98362	0.00000	0.53652	1.89209	1.39094	2.85126	Low	51 - 100 cm	Moderated	Without	Clay-sandy loam
3	14	Dry	CL24	CL24P2	open	66	9	0.54688	0.00000	0.30636	0.00000	0.00000	0.44705	1.89209	1.39094	2.84386	Low	51 - 100 cm	Moderated	Without	Clay-sandy loam
3	14	Dry	CL24	CL24P3	open	71	9	0.41732	0.00000	1.00000	0.00000	0.00000	0.26595	1.89209	1.39094	2.83059	Low	51 - 100 cm	Moderated	Without	Clay-sandy loam
4	17	Rainy	CL3	CL3P1	exclusion	62	14	0.56561	0.20772	0.00000	0.00000	0.03442	0.76583	2.78032	1.39794	2.70728	Medium	21 - 50 cm	Good	Without	Loam
4	17	Rainy	CL3	CL3P2	exclusion	142	20	0.60779	0.36004	0.00000	0.00000	0.04057	0.73233	2.78032	1.39794	2.69955	Medium	21 - 50 cm	Good	Without	Loam
4	17	Rainy	CL3	CL3P3	exclusion	82	15	0.65905	0.32443	0.00000	0.00000	0.05310	0.56828	2.78032	1.39794	2.71912	Medium	21 - 50 cm	Good	Without	Loam
4	17	Rainy	CL6	CL6P1	exclusion	22	1	0.00000	0.23343	0.00000	0.00000	0.05110	0.09773	2.64444	1.40824	2.43057	Medium	0 - 10 cm	Good	Without	Sandy loam
4	17	Rainy	CL6	CL6P2	exclusion	7	3	0.12031	0.06924	0.00000	0.00000	0.06605	0.25003	2.61909	1.41497	2.40929	Medium	0 - 10 cm	Good	Without	Sandy loam
4	17	Rainy	CL6	CL6P3	exclusion	13	1	0.00000	0.12562	0.00000	0.00000	0.05876	0.08515	2.62634	1.41664	2.38998	Medium	0 - 10 cm	Good	Without	Sandy loam
4	17	Rainy	CL7	CL7P1	exclusion	72	5	0.01140	0.44115	0.00000	0.00000	0.05166	0.61218	2.60097	1.41664	2.32720	High	11 - 20 cm	Good	Abundant	Clay-sandy loam
4	17	Rainy	CL7	CL7P2	exclusion	24	4	0.11084	1.00000	0.00000	0.06396	0.04813	0.68053	2.60097	1.41664	2.33612	High	11 - 20 cm	Good	Abundant	Clay-sandy loam
4	17	Rainy	CL7	CL7P3	exclusion	53	8	0.46123	0.37784	0.00000	0.00000	0.03848	0.63038	2.61490	1.41664	2.35626	High	11 - 20 cm	Good	Abundant	Clay-sandy loam
4	17	Rainy	CL8	CL8P1	exclusion	132	8	0.41793	0.14045	0.00000	0.00000	0.02520	0.62758	2.67025	1.40140	2.60576	Medium	21 - 50 cm	Good	Without	Loam
4	17	Rainy	CL8	CL8P2	exclusion	99	13	0.42032	0.15430	0.20372	0.00780	0.03283	0.67353	2.66087	1.40483	2.57317	Medium	21 - 50 cm	Good	Without	Loam
4	17	Rainy	CL8	CL8P3	exclusion	115	13	0.30841	0.16024	0.00000	0.00000	0.02657	0.71990	2.66839	1.40483	2.58200	High	21 - 50 cm	Poorly drained	Few	Clay-sandy loam
4	17	Rainy	CL11	CL11P1	exclusion	86	12	0.13904	0.05836	0.01767	0.00000	0.01326	0.78655	2.76938	1.41162	2.54849	Medium	21 - 50 cm	Good	Without	Loam
4	17	Rainy	CL11	CL11P2	exclusion	44	10	0.51393	0.11078	0.00000	0.00000	0.01371	0.75580	2.76492	1.41162	2.55751	Medium	21 - 50 cm	Good	Without	Loam
4	17	Rainy	CL11	CL11P3	exclusion	89	15	0.52096	0.00000	0.00000	0.00000	0.01604	0.80893	2.76492	1.41162	2.54806	High	21 - 50 cm	Moderated	Without	Loam
4	17	Rainy	CL12	CL12P1	exclusion	65	12	0.52349	0.02374	0.00000	0.00000	0.02082	0.71603	2.74429	1.41664	2.46340	High	51 - 100 cm	Good	Frequent	Loam
4	17	Rainy	CL12	CL12P2	exclusion	88	13	0.26948	0.00000	0.00000	0.04056	0.02448	0.70158	2.74429	1.41664	2.41812	High	51 - 100 cm	Good	Frequent	Loam
4	17	Rainy	CL12	CL12P3	exclusion	101	15	0.53583	0.00000	0.34605	0.18643	0.02773	0.58263	2.74429	1.41664	2.38202	Low	11 - 20 cm	Moderated	Few	Clay loam
4	17	Rainy	CL15	CL15P1	exclusion	65	15	0.57006	0.38477	0.08744	0.00000	0.01088	0.65075	2.78746	1.40654	2.61065	High	> 100 cm	Moderated	Without	Loam
4	17	Rainy	CL15	CL15P2	exclusion	86	13	0.56604	0.12364	0.00000	0.00000	0.01328	0.70950	2.78746	1.40654	2.61507	High	21 - 50 cm	Moderated	Without	Loam
4	17	Rainy	CL15	CL15P3	exclusion	86	17	0.45664	0.26014	0.04620	0.00000	0.00991	0.80575	2.78746	1.40654	2.63075	High	> 100 cm	Moderated	Without	Loam
4	17	Rainy	CL19	CL19P1	exclusion	23	4	0.13413	0.00000	0.00000	0.00000	0.01080	0.61168	3.00260	1.36549	2.92670	Medium	51 - 100 cm	Good	Very few	Loam
4	17	Rainy	CL19	CL19P2	exclusion	18	5	0.38150	0.00000	0.21271	0.00000	0.01089	0.74540	3.00260	1.36549	2.91800	Medium	51 - 100 cm	Good	Very few	Loam
4	17	Rainy	CL19	CL19P3	exclusion	70	13	0.35614	0.00000	0.09767	0.00000	0.01667	0.75230	3.00260	1.36549	2.94979	Medium	51 - 100 cm	Good	Very few	Loam
4	17	Rainy	CL21	CL21P1	exclusion	66	12	0.34217	0.00000	0.00000	0.31123	0.00000	0.75283	2.73719	1.37658	2.85827	Low	51 - 100 cm	Moderated	Without	Clay-sandy loam
4	17	Rainy	CL21	CL21P2	exclusion	23	9	0.37814	0.00000	0.96620	0.00000	0.00000	0.70473	2.73719	1.37658	2.87564	Low	51 - 100 cm	Moderated	Without	Clay-sandy loam
4	17	Rainy	CL21	CL21P3	exclusion	46	6	0.12762	0.00000	0.32620	1.00000	0.00000	0.76800	2.73719	1.37658	2.85087	Low	51 - 100 cm	Moderated	Without	Clay-sandy loam
4	17	Rainy	CL22	CL22P1	exclusion	57	9	0.51078	0.00000	0.52899	0.18019	0.00000	0.79838	2.73719	1.37658	2.87564	Low	51 - 100 cm	Moderated	Without	Clay-sandy loam
4	17	Rainy	CL22	CL22P2	exclusion	35	14	0.75016	0.00000	0.07597	0.42902	0.00000	0.71855	2.73719	1.37658	2.85430	Low	51 - 100 cm	Moderated	Without	Clay-sandy loam
4	17	Rainy	CL22	CL22P3	exclusion	45	6	0.38721	0.00000	0.03504	0.18253	0.00000	0.75030	2.73719	1.37658	2.82674	Low	51 - 100 cm	Moderated	Without	Clay-sandy loam

Measure	Time	Season	Cluster	Cod.plot	Treat	Ind	Spp	Simp	Goats	Cattle	Equine	HPI	Canp	SPrec	MTemp	Alt	OM	SDepth	Drain	Ston	Text
4	17	Rainy	CL23	CL23P1	exclusion	34	4	0.17942	0.00000	0.19721	0.00000	0.00000	0.60893	2.68485	1.38202	2.81358	Low	51 - 100 cm	Moderated	Without	Clay-sandy loam
4	17	Rainy	CL23	CL23P2	exclusion	84	10	0.40188	0.00000	0.31442	0.12715	0.00000	0.59985	2.68485	1.38202	2.81381	Low	51 - 100 cm	Moderated	Without	Clay-sandy loam
4	17	Rainy	CL23	CL23P3	exclusion	109	7	0.48934	0.00000	0.40496	0.00000	0.00000	0.70660	2.68485	1.38202	2.81847	Low	51 - 100 cm	Moderated	Without	Clay-sandy loam
4	17	Rainy	CL24	CL24P1	exclusion	411	6	0.00007	0.00000	0.00000	0.98362	0.00000	0.75795	2.70243	1.37658	2.85126	Low	51 - 100 cm	Moderated	Without	Clay-sandy loam
4	17	Rainy	CL24	CL24P2	exclusion	156	9	0.43008	0.00000	0.30636	0.00000	0.00000	0.74030	2.70243	1.37658	2.84386	Low	51 - 100 cm	Moderated	Without	Clay-sandy loam
4	17	Rainy	CL24	CL24P3	exclusion	66	6	0.02578	0.00000	1.00000	0.00000	0.00000	0.75603	2.69897	1.37658	2.83059	Low	51 - 100 cm	Moderated	Without	Clay-sandy loam
4	17	Rainy	CL3	CL3P1	open	130	16	0.22662	0.20772	0.00000	0.00000	0.03442	0.71515	2.78032	1.39794	2.70728	Medium	21 - 50 cm	Good	Without	Loam
4	17	Rainy	CL3	CL3P2	open	66	15	0.69209	0.36004	0.00000	0.00000	0.04057	0.81495	2.78032	1.39794	2.69955	Medium	21 - 50 cm	Good	Without	Loam
4	17	Rainy	CL3	CL3P3	open	135	18	0.58472	0.32443	0.00000	0.00000	0.05310	0.62510	2.78032	1.39794	2.71912	Medium	21 - 50 cm	Good	Without	Loam
4	17	Rainy	CL6	CL6P1	open	26	3	0.00204	0.23343	0.00000	0.00000	0.05110	0.15363	2.64444	1.40824	2.43057	Medium	0 - 10 cm	Good	Without	Sandy loam
4	17	Rainy	CL6	CL6P2	open	64	1	0.00000	0.06924	0.00000	0.00000	0.06605	0.06750	2.61909	1.41497	2.40929	Medium	0 - 10 cm	Good	Without	Sandy loam
4	17	Rainy	CL6	CL6P3	open	19	2	0.05366	0.12562	0.00000	0.00000	0.05876	0.11545	2.62634	1.41664	2.38998	Medium	0 - 10 cm	Good	Without	Sandy loam
4	17	Rainy	CL7	CL7P1	open	36	3	0.01101	0.44115	0.00000	0.00000	0.05166	0.56403	2.60097	1.41664	2.32720	High	11 - 20 cm	Good	Abundant	Clay-sandy loam
4	17	Rainy	CL7	CL7P2	open	62	5	0.00935	1.00000	0.00000	0.06396	0.04813	0.33560	2.60097	1.41664	2.33612	High	11 - 20 cm	Good	Abundant	Clay-sandy loam
4	17	Rainy	CL7	CL7P3	open	21	5	0.33222	0.37784	0.00000	0.00000	0.03848	0.68780	2.61490	1.41664	2.35626	High	11 - 20 cm	Good	Abundant	Clay-sandy loam
4	17	Rainy	CL8	CL8P1	open	124	12	0.34066	0.14045	0.00000	0.00000	0.02520	0.61515	2.67025	1.40140	2.60576	Medium	21 - 50 cm	Good	Without	Loam
4	17	Rainy	CL8	CL8P2	open	142	13	0.43977	0.15430	0.20372	0.00780	0.03283	0.46080	2.66087	1.40483	2.57317	Medium	21 - 50 cm	Good	Without	Loam
4	17	Rainy	CL8	CL8P3	open	43	6	0.18453	0.16024	0.00000	0.00000	0.02657	0.67270	2.66839	1.40483	2.58200	High	21 - 50 cm	Poorly drained	Few	Clay-sandy loam
4	17	Rainy	CL11	CL11P1	open	50	11	0.41427	0.05836	0.01767	0.00000	0.01326	0.80360	2.76938	1.41162	2.54849	Medium	21 - 50 cm	Good	Without	Loam
4	17	Rainy	CL11	CL11P2	open	173	11	0.34919	0.11078	0.00000	0.00000	0.01371	0.78225	2.76492	1.41162	2.55751	Medium	21 - 50 cm	Good	Without	Loam
4	17	Rainy	CL11	CL11P3	open	22	9	0.58386	0.00000	0.00000	0.00000	0.01604	0.70743	2.76492	1.41162	2.54806	High	21 - 50 cm	Moderated	Without	Loam
4	17	Rainy	CL12	CL12P1	open	68	9	0.13609	0.02374	0.00000	0.00000	0.02082	0.73198	2.74429	1.41664	2.46340	High	51 - 100 cm	Good	Frequent	Loam
4	17	Rainy	CL12	CL12P2	open	27	7	0.43909	0.00000	0.00000	0.04056	0.02448	0.61255	2.74429	1.41664	2.41812	High	51 - 100 cm	Good	Frequent	Loam
4	17	Rainy	CL12	CL12P3	open	48	8	0.56199	0.00000	0.34605	0.18643	0.02773	0.37900	2.74429	1.41664	2.38202	Low	11 - 20 cm	Moderated	Few	Clay loam
4	17	Rainy	CL15	CL15P1	open	119	13	0.35578	0.38477	0.08744	0.00000	0.01088	0.77295	2.78746	1.40654	2.61065	High	> 100 cm	Moderated	Without	Loam
4	17	Rainy	CL15	CL15P2	open	100	16	0.50707	0.12364	0.00000	0.00000	0.01328	0.71863	2.78746	1.40654	2.61507	High	21 - 50 cm	Moderated	Without	Loam
4	17	Rainy	CL15	CL15P3	open	74	17	0.67174	0.26014	0.04620	0.00000	0.00991	0.87275	2.78746	1.40654	2.63075	High	> 100 cm	Moderated	Without	Loam
4	17	Rainy	CL19	CL19P1	open	17	7	0.64577	0.00000	0.00000	0.00000	0.01080	0.58868	3.00260	1.36549	2.92670	Medium	51 - 100 cm	Good	Very few	Loam
4	17	Rainy	CL19	CL19P2	open	24	5	0.06536	0.00000	0.21271	0.00000	0.01089	0.65015	3.00260	1.36549	2.91800	Medium	51 - 100 cm	Good	Very few	Loam
4	17	Rainy	CL19	CL19P3	open	32	6	0.32829	0.00000	0.09767	0.00000	0.01667	0.64088	3.00260	1.36549	2.94979	Medium	51 - 100 cm	Good	Very few	Loam
4	17	Rainy	CL21	CL21P1	open	54	11	0.49432	0.00000	0.00000	0.31123	0.00000	0.78733	2.73719	1.37658	2.85827	Low	51 - 100 cm	Moderated	Without	Clay-sandy loam
4	17	Rainy	CL21	CL21P2	open	12	4	0.22758	0.00000	0.96620	0.00000	0.00000	0.69090	2.73719	1.37658	2.87564	Low	51 - 100 cm	Moderated	Without	Clay-sandy loam
4	17	Rainy	CL21	CL21P3	open	34	11	0.69042	0.00000	0.32620	1.00000	0.00000	0.76613	2.73719	1.37658	2.85087	Low	51 - 100 cm	Moderated	Without	Clay-sandy loam
4	17	Rainy	CL22	CL22P1	open	41	8	0.41309	0.00000	0.52899	0.18019	0.00000	0.77420	2.73719	1.37658	2.87564	Low	51 - 100 cm	Moderated	Without	Clay-sandy loam
4	17	Rainy	CL22	CL22P2	open	40	10	0.28560	0.00000	0.07597	0.42902	0.00000	0.72450	2.73719	1.37658	2.85430	Low	51 - 100 cm	Moderated	Without	Clay-sandy loam
4	17	Rainy	CL22	CL22P3	open	31	8	0.54346	0.00000	0.03504	0.18253	0.00000	0.76255	2.73719	1.37658	2.82674	Low	51 - 100 cm	Moderated	Without	Clay-sandy loam
4	17	Rainy	CL23	CL23P1	open	31	6	0.29108	0.00000	0.19721	0.00000	0.00000	0.67263	2.68485	1.38202	2.81358	Low	51 - 100 cm	Moderated	Without	Clay-sandy loam
4	17	Rainy	CL23	CL23P2	open	125	13	0.15326	0.00000	0.31442	0.12715	0.00000	0.67373	2.68485	1.38202	2.81381	Low	51 - 100 cm	Moderated	Without	Clay-sandy loam
4	17	Rainy	CL23	CL23P3	open	107	11	0.44935	0.00000	0.40496	0.00000	0.00000	0.69535	2.68485	1.38202	2.81847	Low	51 - 100 cm	Moderated	Without	Clay-sandy loam
4	17	Rainy	CL24	CL24P1	open	68	11	0.25396	0.00000	0.00000	0.98362	0.00000	0.76983	2.70243	1.37658	2.85126	Low	51 - 100 cm	Moderated	Without	Clay-sandy loam
4	17	Rainy	CL24	CL24P2	open	60	9	0.52634	0.00000	0.30636	0.00000	0.00000	0.68478	2.70243	1.37658	2.84386	Low	51 - 100 cm	Moderated	Without	Clay-sandy loam
4	17	Rainy	CL24	CL24P3	open	68	9	0.40531	0.00000	1.00000	0.00000	0.00000	0.65275	2.69897	1.37658	2.83059	Low	51 - 100 cm	Moderated	Without	Clay-sandy loam
5	23	Dry	CL3	CL3P1	exclusion	58	15	0.57095	0.20772	0.00000	0.00000	0.03442	0.58740	1.97772	1.38561	2.70728	Medium	21 - 50 cm	Good	Without	Loam
5	23	Dry	CL3	CL3P2	exclusion	127	17	0.60620	0.36004	0.00000	0.00000	0.04057	0.55635	1.97772	1.38561	2.69955	Medium	21 - 50 cm	Good	Without	Loam
5	23	Dry	CL3	CL3P3	exclusion	75	15	0.66591	0.32443	0.00000	0.00000	0.05310	0.34878	1.97772	1.38561	2.71912	Medium	21 - 50 cm	Good	Without	Loam
5	23	Dry	CL6	CL6P1	exclusion	13	1	0.00000	0.23343	0.00000	0.00000	0.05110	0.03913	1.64345	1.39270	2.43057	Medium	0 - 10 cm	Good	Without	Sandy loam
5	23	Dry	CL6	CL6P2	exclusion	4	1	0.00000	0.06924	0.00000	0.00000	0.06605	0.18193	1.63347	1.39620	2.40929	Medium	0 - 10 cm	Good	Without	Sandy loam
5	23	Dry	CL6	CL6P3	exclusion	13	1	0.00000	0.12562	0.00000	0.00000	0.05876	0.05578	1.64345	1.39620	2.38998	Medium	0 - 10 cm	Good	Without	Sandy loam
5	23	Dry	CL7	CL7P1	exclusion	49	3	0.00451	0.44115	0.00000	0.00000	0.05166	0.37645	1.65321	1.39794	2.32720	High	11 - 20 cm	Good	Abundant	Clay-sandy loam
5	23	Dry	CL7	CL7P2	exclusion	31	3	0.06950	1.00000	0.00000	0.06396	0.04813	0.29590	1.65321	1.39794	2.33612	High	11 - 20 cm	Good	Abundant	Clay-sandy loam
5	23	Dry	CL7	CL7P3	exclusion	33	4	0.21242	0.37784	0.00000	0.00000	0.03848	0.39880	1.66276	1.39620	2.35626	High	11 - 20 cm	Good	Abundant	Clay-sandy loam
5	23	Dry	CL8	CL8P1	exclusion	132	8	0.42311	0.14045	0.00000	0.00000	0.02520	0.60438	1.84510	1.38382	2.60576	Medium	21 - 50 cm	Good	Without	Loam
5	23	Dry	CL8	CL8P2	exclusion	82	13	0.49121	0.15430	0.20372	0.00780	0.03283	0.55603	1.84510	1.38561	2.57317	Medium	21 - 50 cm	Good	Without	Loam

Measure	Time	Season	Cluster	Cod.plot	Treat	Ind	Spp	Simp	Goats	Cattle	Equine	HPI	Canp	SPrec	MTemp	Alt	OM	SDepth	Drain	Ston	Text
5	23	Dry	CL8	CL8P3	exclusion	105	13	0.32806	0.16024	0.00000	0.00000	0.02657	0.46150	1.86332	1.38561	2.58200	High	21 - 50 cm	Poorly drained	Few	Clay-sandy loam
5	23	Dry	CL11	CL11P1	exclusion	85	12	0.14316	0.05836	0.01767	0.00000	0.01326	0.61230	1.88649	1.39794	2.54849	Medium	21 - 50 cm	Good	Without	Loam
5	23	Dry	CL11	CL11P2	exclusion	40	10	0.51517	0.11078	0.00000	0.00000	0.01371	0.54073	1.89209	1.39794	2.55751	Medium	21 - 50 cm	Good	Without	Loam
5	23	Dry	CL11	CL11P3	exclusion	87	15	0.51508	0.00000	0.00000	0.00000	0.01604	0.40843	1.89209	1.39794	2.54806	High	21 - 50 cm	Moderated	Without	Loam
5	23	Dry	CL12	CL12P1	exclusion	50	11	0.59610	0.02374	0.00000	0.00000	0.02082	0.26463	2.33846	1.39967	2.46340	High	51 - 100 cm	Good	Frequent	Loam
5	23	Dry	CL12	CL12P2	exclusion	55	13	0.41348	0.00000	0.00000	0.04056	0.02448	0.29288	2.34044	1.39967	2.41812	High	51 - 100 cm	Good	Frequent	Loam
5	23	Dry	CL12	CL12P3	exclusion	89	15	0.53382	0.00000	0.34605	0.18643	0.02773	0.23843	2.33445	1.39967	2.38202	Low	11 - 20 cm	Moderated	Few	Clay loam
5	23	Dry	CL15	CL15P1	exclusion	64	15	0.56121	0.38477	0.08744	0.00000	0.01088	0.52773	1.95904	1.39270	2.61065	High	> 100 cm	Moderated	Without	Loam
5	23	Dry	CL15	CL15P2	exclusion	63	13	0.51100	0.12364	0.00000	0.00000	0.01328	0.45038	1.95904	1.39270	2.61507	High	21 - 50 cm	Moderated	Without	Loam
5	23	Dry	CL15	CL15P3	exclusion	86	17	0.47082	0.26014	0.04620	0.00000	0.00991	0.58673	1.95904	1.39270	2.63075	High	> 100 cm	Moderated	Without	Loam
5	23	Dry	CL19	CL19P1	exclusion	19	4	0.11781	0.00000	0.00000	0.00000	0.01080	0.44185	2.36549	1.37475	2.92670	Medium	51 - 100 cm	Good	Very few	Loam
5	23	Dry	CL19	CL19P2	exclusion	16	5	0.41888	0.00000	0.21271	0.00000	0.01089	0.58910	2.36549	1.37475	2.91800	Medium	51 - 100 cm	Good	Very few	Loam
5	23	Dry	CL19	CL19P3	exclusion	54	13	0.35718	0.00000	0.09767	0.00000	0.01667	0.65665	2.36736	1.37475	2.94979	Medium	51 - 100 cm	Good	Very few	Loam
5	23	Dry	CL21	CL21P1	exclusion	64	13	0.35228	0.00000	0.00000	0.31123	0.00000	0.59710	1.94939	1.35793	2.85827	Low	51 - 100 cm	Moderated	Without	Clay-sandy loam
5	23	Dry	CL21	CL21P2	exclusion	28	11	0.44895	0.00000	0.96620	0.00000	0.00000	0.65285	1.95424	1.35793	2.87564	Low	51 - 100 cm	Moderated	Without	Clay-sandy loam
5	23	Dry	CL21	CL21P3	exclusion	54	9	0.19820	0.00000	0.32620	1.00000	0.00000	0.57120	1.94939	1.35793	2.85087	Low	51 - 100 cm	Moderated	Without	Clay-sandy loam
5	23	Dry	CL22	CL22P1	exclusion	54	8	0.49291	0.00000	0.52899	0.18019	0.00000	0.72773	1.95424	1.35793	2.87564	Low	51 - 100 cm	Moderated	Without	Clay-sandy loam
5	23	Dry	CL22	CL22P2	exclusion	31	14	0.78578	0.00000	0.07597	0.42902	0.00000	0.65948	1.94939	1.35793	2.85430	Low	51 - 100 cm	Moderated	Without	Clay-sandy loam
5	23	Dry	CL22	CL22P3	exclusion	34	6	0.27682	0.00000	0.03504	0.18253	0.00000	0.65170	1.94448	1.35793	2.82674	Low	51 - 100 cm	Moderated	Without	Clay-sandy loam
5	23	Dry	CL23	CL23P1	exclusion	32	4	0.19693	0.00000	0.19721	0.00000	0.00000	0.27673	1.87506	1.36173	2.81358	Low	51 - 100 cm	Moderated	Without	Clay-sandy loam
5	23	Dry	CL23	CL23P2	exclusion	76	10	0.35369	0.00000	0.31442	0.12715	0.00000	0.30560	1.87506	1.36173	2.81381	Low	51 - 100 cm	Moderated	Without	Clay-sandy loam
5	23	Dry	CL23	CL23P3	exclusion	89	7	0.50102	0.00000	0.40496	0.00000	0.00000	0.40588	1.87506	1.36173	2.81847	Low	51 - 100 cm	Moderated	Without	Clay-sandy loam
5	23	Dry	CL24	CL24P1	exclusion	403	4	0.00000	0.00000	0.00000	0.98362	0.00000	0.57357	1.89209	1.35793	2.85126	Low	51 - 100 cm	Moderated	Without	Clay-sandy loam
5	23	Dry	CL24	CL24P2	exclusion	149	10	0.39110	0.00000	0.30636	0.00000	0.00000	0.47620	1.89209	1.35793	2.84386	Low	51 - 100 cm	Moderated	Without	Clay-sandy loam
5	23	Dry	CL24	CL24P3	exclusion	64	7	0.02846	0.00000	1.00000	0.00000	0.00000	0.47125	1.89209	1.35984	2.83059	Low	51 - 100 cm	Moderated	Without	Clay-sandy loam
5	23	Dry	CL3	CL3P1	open	123	14	0.20086	0.20772	0.00000	0.00000	0.03442	0.51790	1.97772	1.38561	2.70728	Medium	21 - 50 cm	Good	Without	Loam
5	23	Dry	CL3	CL3P2	open	67	15	0.65386	0.36004	0.00000	0.00000	0.04057	0.51560	1.97772	1.38561	2.69955	Medium	21 - 50 cm	Good	Without	Loam
5	23	Dry	CL3	CL3P3	open	116	17	0.56271	0.32443	0.00000	0.00000	0.05310	0.48953	1.97772	1.38561	2.71912	Medium	21 - 50 cm	Good	Without	Loam
5	23	Dry	CL6	CL6P1	open	20	3	0.00471	0.23343	0.00000	0.00000	0.05110	0.13880	1.64345	1.39270	2.43057	Medium	0 - 10 cm	Good	Without	Sandy loam
5	23	Dry	CL6	CL6P2	open	35	1	0.00000	0.06924	0.00000	0.00000	0.06605	0.05618	1.63347	1.39620	2.40929	Medium	0 - 10 cm	Good	Without	Sandy loam
5	23	Dry	CL6	CL6P3	open	13	1	0.00000	0.12562	0.00000	0.00000	0.05876	0.06130	1.64345	1.39620	2.38998	Medium	0 - 10 cm	Good	Without	Sandy loam
5	23	Dry	CL7	CL7P1	open	32	2	0.00099	0.44115	0.00000	0.00000	0.05166	0.36963	1.65321	1.39794	2.32720	High	11 - 20 cm	Good	Abundant	Clay-sandy loam
5	23	Dry	CL7	CL7P2	open	29	3	0.00489	1.00000	0.00000	0.06396	0.04813	0.16270	1.65321	1.39794	2.33612	High	11 - 20 cm	Good	Abundant	Clay-sandy loam
5	23	Dry	CL7	CL7P3	open	13	2	0.13168	0.37784	0.00000	0.00000	0.03848	0.35183	1.66276	1.39620	2.35626	High	11 - 20 cm	Good	Abundant	Clay-sandy loam
5	23	Dry	CL8	CL8P1	open	121	13	0.34677	0.14045	0.00000	0.00000	0.02520	0.47975	1.84510	1.38382	2.60576	Medium	21 - 50 cm	Good	Without	Loam
5	23	Dry	CL8	CL8P2	open	130	11	0.41664	0.15430	0.20372	0.00780	0.03283	0.32945	1.84510	1.38561	2.57317	Medium	21 - 50 cm	Good	Without	Loam
5	23	Dry	CL8	CL8P3	open	39	6	0.18006	0.16024	0.00000	0.00000	0.02657	0.62873	1.86332	1.38561	2.58200	High	21 - 50 cm	Poorly drained	Few	Clay-sandy loam
5	23	Dry	CL11	CL11P1	open	46	11	0.41974	0.05836	0.01767	0.00000	0.01326	0.65428	1.88649	1.39794	2.54849	Medium	21 - 50 cm	Good	Without	Loam
5	23	Dry	CL11	CL11P2	open	168	11	0.34199	0.11078	0.00000	0.00000	0.01371	0.53023	1.89209	1.39794	2.55751	Medium	21 - 50 cm	Good	Without	Loam
5	23	Dry	CL11	CL11P3	open	21	9	0.57127	0.00000	0.00000	0.00000	0.01604	0.49678	1.89209	1.39794	2.54806	High	21 - 50 cm	Moderated	Without	Loam
5	23	Dry	CL12	CL12P1	open	67	9	0.12544	0.02374	0.00000	0.00000	0.02082	0.30320	2.33846	1.39967	2.46340	High	51 - 100 cm	Good	Frequent	Loam
5	23	Dry	CL12	CL12P2	open	25	7	0.44944	0.00000	0.00000	0.04056	0.02448	0.40963	2.34044	1.39967	2.41812	High	51 - 100 cm	Good	Frequent	Loam
5	23	Dry	CL12	CL12P3	open	45	9	0.59995	0.00000	0.34605	0.18643	0.02773	0.16573	2.33445	1.39967	2.38202	Low	11 - 20 cm	Moderated	Few	Clay loam
5	23	Dry	CL15	CL15P1	open	114	13	0.36404	0.38477	0.08744	0.00000	0.01088	0.48650	1.95904	1.39270	2.61065	High	> 100 cm	Moderated	Without	Loam
5	23	Dry	CL15	CL15P2	open	87	15	0.46036	0.12364	0.00000	0.00000	0.01328	0.46960	1.95904	1.39270	2.61507	High	21 - 50 cm	Moderated	Without	Loam
5	23	Dry	CL15	CL15P3	open	68	16	0.64869	0.26014	0.04620	0.00000	0.00991	0.60778	1.95904	1.39270	2.63075	High	> 100 cm	Moderated	Without	Loam
5	23	Dry	CL19	CL19P1	open	16	7	0.66613	0.00000	0.00000	0.00000	0.01080	0.35413	2.36549	1.37475	2.92670	Medium	51 - 100 cm	Good	Very few	Loam
5	23	Dry	CL19	CL19P2	open	20	4	0.06380	0.00000	0.21271	0.00000	0.01089	0.62780	2.36549	1.37475	2.91800	Medium	51 - 100 cm	Good	Very few	Loam
5	23	Dry	CL19	CL19P3	open	29	6	0.35922	0.00000	0.09767	0.00000	0.01667	0.60883	2.36736	1.37475	2.94979	Medium	51 - 100 cm	Good	Very few	Loam
5	23	Dry	CL21	CL21P1	open	50	12	0.48962	0.00000	0.00000	0.31123	0.00000	0.55740	1.94939	1.35793	2.85827	Low	51 - 100 cm	Moderated	Without	Clay-sandy loam
5	23	Dry	CL21	CL21P2	open	11	4	0.27301	0.00000	0.96620	0.00000	0.00000	0.58253	1.95424	1.35793	2.87564	Low	51 - 100 cm	Moderated	Without	Clay-sandy loam
5	23	Dry	CL21	CL21P3	open	32	11	0.73236	0.00000	0.32620	1.00000	0.00000	0.57465	1.94939	1.35793	2.85087	Low	51 - 100 cm	Moderated	Without	Clay-sandy loam
5	23	Dry	CL22	CL22P1	open	41	8	0.41093	0.00000	0.52899	0.18019	0.00000	0.62520	1.95424	1.35793	2.87564	Low	51 - 100 cm	Moderated	Without	Clay-sandy loam

Measure	Time	Season	Cluster	Cod.plot	Treat	Ind	Spp	Simp	Goats	Cattle	Equine	HPI	Canp	SPrec	MTemp	Alt	OM	SDepth	Drain	Ston	Text
5	23	Dry	CL22	CL22P2	open	37	10	0.29340	0.00000	0.07597	0.42902	0.00000	0.64065	1.94939	1.35793	2.85430	Low	51 - 100 cm	Moderated	Without	Clay-sandy loam
5	23	Dry	CL22	CL22P3	open	30	8	0.54298	0.00000	0.03504	0.18253	0.00000	0.63193	1.94448	1.35793	2.82674	Low	51 - 100 cm	Moderated	Without	Clay-sandy loam
5	23	Dry	CL23	CL23P1	open	27	6	0.34225	0.00000	0.19721	0.00000	0.00000	0.33094	1.87506	1.36173	2.81358	Low	51 - 100 cm	Moderated	Without	Clay-sandy loam
5	23	Dry	CL23	CL23P2	open	116	13	0.11965	0.00000	0.31442	0.12715	0.00000	0.40118	1.87506	1.36173	2.81381	Low	51 - 100 cm	Moderated	Without	Clay-sandy loam
5	23	Dry	CL23	CL23P3	open	99	11	0.40499	0.00000	0.40496	0.00000	0.00000	0.40533	1.87506	1.36173	2.81847	Low	51 - 100 cm	Moderated	Without	Clay-sandy loam
5	23	Dry	CL24	CL24P1	open	61	7	0.16467	0.00000	0.00000	0.98362	0.00000	0.53652	1.89209	1.35793	2.85126	Low	51 - 100 cm	Moderated	Without	Clay-sandy loam
5	23	Dry	CL24	CL24P2	open	52	9	0.46131	0.00000	0.30636	0.00000	0.00000	0.44705	1.89209	1.35793	2.84386	Low	51 - 100 cm	Moderated	Without	Clay-sandy loam
5	23	Dry	CL24	CL24P3	open	64	9	0.40259	0.00000	1.00000	0.00000	0.00000	0.26595	1.89209	1.35984	2.83059	Low	51 - 100 cm	Moderated	Without	Clay-sandy loam

Supplementary Table S6. Database to evaluate the dynamics of natural regeneration.

Measure	Cluster	Cod.Plot	Formation	Density	Mort	Recr	Time	Season	Treat	SPrec
3	CL3	CL3P1	semideciduous	dense	6.66667	8.19672	14	Dry	exclusion	1.97772
3	CL3	CL3P2	semideciduous	dense	12.84404	21.48760	14	Dry	exclusion	1.97772
3	CL3	CL3P3	semideciduous	dense	38.23529	10.00000	14	Dry	exclusion	1.97772
3	CL6	CL6P1	deciduous	sparse	33.33333	23.07692	14	Dry	exclusion	1.64345
3	CL6	CL6P2	deciduous	sparse	87.50000	75.00000	14	Dry	exclusion	1.63347
3	CL6	CL6P3	deciduous	sparse	63.63636	42.85714	14	Dry	exclusion	1.64345
3	CL7	CL7P1	deciduous	sparse	84.61538	79.10448	14	Dry	exclusion	1.65321
3	CL7	CL7P2	deciduous	sparse	63.04348	19.04762	14	Dry	exclusion	1.65321
3	CL7	CL7P3	deciduous	sparse	45.45455	12.50000	14	Dry	exclusion	1.66276
3	CL8	CL8P1	deciduous	dense	8.33333	49.07407	14	Dry	exclusion	1.84510
3	CL8	CL8P2	deciduous	dense	17.94872	51.51515	14	Dry	exclusion	1.84510
3	CL8	CL8P3	deciduous	dense	3.70370	75.92593	14	Dry	exclusion	1.86332
3	CL11	CL11P1	deciduous	dense	4.59770	0.00000	14	Dry	exclusion	1.88649
3	CL11	CL11P2	deciduous	dense	32.25806	0.00000	14	Dry	exclusion	1.89209
3	CL11	CL11P3	deciduous	dense	13.04348	1.23457	14	Dry	exclusion	1.89209
3	CL12	CL12P1	deciduous	semidense	73.97260	43.28358	14	Dry	exclusion	2.33846
3	CL12	CL12P2	deciduous	semidense	32.05128	58.26772	14	Dry	exclusion	2.34044
3	CL12	CL12P3	deciduous	semidense	35.25180	4.25532	14	Dry	exclusion	2.33445
3	CL15	CL15P1	deciduous	semidense	34.72222	6.00000	14	Dry	exclusion	1.95904
3	CL15	CL15P2	deciduous	semidense	16.36364	8.00000	14	Dry	exclusion	1.95904
3	CL15	CL15P3	deciduous	semidense	4.28571	8.21918	14	Dry	exclusion	1.95904
3	CL19	CL19P1	semideciduous	semidense	17.85714	4.16667	14	Dry	exclusion	2.36549
3	CL19	CL19P2	semideciduous	semidense	11.76471	16.66667	14	Dry	exclusion	2.36549
3	CL19	CL19P3	semideciduous	semidense	12.50000	15.51724	14	Dry	exclusion	2.36736
3	CL21	CL21P1	semideciduous	dense	11.47541	14.28571	14	Dry	exclusion	1.94939
3	CL21	CL21P2	semideciduous	dense	34.61538	32.00000	14	Dry	exclusion	1.95424
3	CL21	CL21P3	semideciduous	dense	24.52830	4.76190	14	Dry	exclusion	1.94939
3	CL22	CL22P1	semideciduous	dense	23.80952	5.88235	14	Dry	exclusion	1.95424
3	CL22	CL22P2	semideciduous	dense	22.85714	18.18182	14	Dry	exclusion	1.94939
3	CL22	CL22P3	semideciduous	dense	38.33333	11.90476	14	Dry	exclusion	1.94448
3	CL23	CL23P1	semideciduous	sparse	3.84615	26.47059	14	Dry	exclusion	1.87506
3	CL23	CL23P2	semideciduous	sparse	17.52577	1.23457	14	Dry	exclusion	1.87506
3	CL23	CL23P3	semideciduous	sparse	34.96933	3.63636	14	Dry	exclusion	1.87506
3	CL24	CL24P1	semideciduous	sparse	10.71429	2.91262	14	Dry	exclusion	1.89209
3	CL24	CL24P2	semideciduous	sparse	55.28701	5.12821	14	Dry	exclusion	1.89209
3	CL24	CL24P3	semideciduous	sparse	25.97403	9.52381	14	Dry	exclusion	1.89209
3	CL3	CL3P1	semideciduous	dense	11.51079	5.38462	14	Dry	open	1.97772
3	CL3	CL3P2	semideciduous	dense	16.12903	18.75000	14	Dry	open	1.97772
3	CL3	CL3P3	semideciduous	dense	17.39130	8.80000	14	Dry	open	1.97772
3	CL6	CL6P1	deciduous	sparse	41.17647	52.38095	14	Dry	open	1.64345
3	CL6	CL6P2	deciduous	sparse	56.19048	8.00000	14	Dry	open	1.63347
3	CL6	CL6P3	deciduous	sparse	60.71429	21.42857	14	Dry	open	1.64345
3	CL7	CL7P1	deciduous	sparse	82.60870	75.00000	14	Dry	open	1.65321
3	CL7	CL7P2	deciduous	sparse	61.94690	15.68627	14	Dry	open	1.65321
3	CL7	CL7P3	deciduous	sparse	75.92593	23.52941	14	Dry	open	1.66276
3	CL8	CL8P1	deciduous	dense	5.00000	30.90909	14	Dry	open	1.84510
3	CL8	CL8P2	deciduous	dense	7.96460	22.96296	14	Dry	open	1.84510
3	CL8	CL8P3	deciduous	dense	17.24138	41.46341	14	Dry	open	1.86332
3	CL11	CL11P1	deciduous	dense	19.04762	5.55556	14	Dry	open	1.88649
3	CL11	CL11P2	deciduous	dense	22.22222	0.00000	14	Dry	open	1.89209
3	CL11	CL11P3	deciduous	dense	39.28571	0.00000	14	Dry	open	1.89209
3	CL12	CL12P1	deciduous	semidense	9.72222	5.79710	14	Dry	open	2.33846
3	CL12	CL12P2	deciduous	semidense	40.47619	13.79310	14	Dry	open	2.34044
3	CL12	CL12P3	deciduous	semidense	70.94017	34.61538	14	Dry	open	2.33445
3	CL15	CL15P1	deciduous	semidense	6.79612	3.03030	14	Dry	open	1.95904
3	CL15	CL15P2	deciduous	semidense	6.06061	16.21622	14	Dry	open	1.95904
3	CL15	CL15P3	deciduous	semidense	21.53846	13.55932	14	Dry	open	1.95904
3	CL19	CL19P1	semideciduous	semidense	22.72727	19.04762	14	Dry	open	2.36549
3	CL19	CL19P2	semideciduous	semidense	27.58621	12.50000	14	Dry	open	2.36549
3	CL19	CL19P3	semideciduous	semidense	10.00000	0.00000	14	Dry	open	2.36736
3	CL21	CL21P1	semideciduous	dense	27.53623	7.40741	14	Dry	open	1.94939
3	CL21	CL21P2	semideciduous	dense	85.29412	44.44444	14	Dry	open	1.95424
3	CL21	CL21P3	semideciduous	dense	41.53846	5.00000	14	Dry	open	1.94939
3	CL22	CL22P1	semideciduous	dense	26.31579	12.50000	14	Dry	open	1.95424
3	CL22	CL22P2	semideciduous	dense	26.00000	11.90476	14	Dry	open	1.94939

Measure	Cluster	Cod.Plot	Formation	Density	Mort	Recr	Time	Season	Treat	SPrec
3	CL22	CL22P3	semideciduous	dense	32.50000	0.00000	14	Dry	open	1.94448
3	CL23	CL23P1	semideciduous	sparse	24.24242	28.57143	14	Dry	open	1.87506
3	CL23	CL23P2	semideciduous	sparse	75.48263	0.78125	14	Dry	open	1.87506
3	CL23	CL23P3	semideciduous	sparse	35.95506	3.38983	14	Dry	open	1.87506
3	CL24	CL24P1	semideciduous	sparse	48.93617	15.78947	14	Dry	open	1.89209
3	CL24	CL24P2	semideciduous	sparse	75.18797	0.00000	14	Dry	open	1.89209
3	CL24	CL24P3	semideciduous	sparse	39.65517	1.40845	14	Dry	open	1.89209
4	CL3	CL3P1	semideciduous	dense	3.27869	4.83871	17	Rainy	exclusion	2.78032
4	CL3	CL3P2	semideciduous	dense	5.78512	19.71831	17	Rainy	exclusion	2.78032
4	CL3	CL3P3	semideciduous	dense	10.00000	23.17073	17	Rainy	exclusion	2.78032
4	CL6	CL6P1	deciduous	sparse	0.00000	40.90909	17	Rainy	exclusion	2.64444
4	CL6	CL6P2	deciduous	sparse	0.00000	42.85714	17	Rainy	exclusion	2.61909
4	CL6	CL6P3	deciduous	sparse	0.00000	46.15385	17	Rainy	exclusion	2.62634
4	CL7	CL7P1	deciduous	sparse	7.46269	13.88889	17	Rainy	exclusion	2.60097
4	CL7	CL7P2	deciduous	sparse	0.00000	12.50000	17	Rainy	exclusion	2.60097
4	CL7	CL7P3	deciduous	sparse	31.25000	37.73585	17	Rainy	exclusion	2.61490
4	CL8	CL8P1	deciduous	dense	0.00000	18.18182	17	Rainy	exclusion	2.67025
4	CL8	CL8P2	deciduous	dense	3.03030	35.35354	17	Rainy	exclusion	2.66087
4	CL8	CL8P3	deciduous	dense	0.92593	6.95652	17	Rainy	exclusion	2.66839
4	CL11	CL11P1	deciduous	dense	1.20482	4.65116	17	Rainy	exclusion	2.76938
4	CL11	CL11P2	deciduous	dense	19.04762	22.72727	17	Rainy	exclusion	2.76492
4	CL11	CL11P3	deciduous	dense	3.70370	12.35955	17	Rainy	exclusion	2.76492
4	CL12	CL12P1	deciduous	semidense	14.92537	12.30769	17	Rainy	exclusion	2.74429
4	CL12	CL12P2	deciduous	semidense	34.64567	5.68182	17	Rainy	exclusion	2.74429
4	CL12	CL12P3	deciduous	semidense	3.19149	9.90099	17	Rainy	exclusion	2.74429
4	CL15	CL15P1	deciduous	semidense	2.00000	24.61538	17	Rainy	exclusion	2.78746
4	CL15	CL15P2	deciduous	semidense	2.00000	43.02326	17	Rainy	exclusion	2.78746
4	CL15	CL15P3	deciduous	semidense	0.00000	15.11628	17	Rainy	exclusion	2.78746
4	CL19	CL19P1	semideciduous	semidense	8.33333	4.34783	17	Rainy	exclusion	3.00260
4	CL19	CL19P2	semideciduous	semidense	0.00000	0.00000	17	Rainy	exclusion	3.00260
4	CL19	CL19P3	semideciduous	semidense	5.17241	21.42857	17	Rainy	exclusion	3.00260
4	CL21	CL21P1	semideciduous	dense	0.00000	4.54545	17	Rainy	exclusion	2.73719
4	CL21	CL21P2	semideciduous	dense	20.00000	13.04348	17	Rainy	exclusion	2.73719
4	CL21	CL21P3	semideciduous	dense	7.14286	15.21739	17	Rainy	exclusion	2.73719
4	CL22	CL22P1	semideciduous	dense	3.92157	14.03509	17	Rainy	exclusion	2.73719
4	CL22	CL22P2	semideciduous	dense	9.09091	14.28571	17	Rainy	exclusion	2.73719
4	CL22	CL22P3	semideciduous	dense	7.14286	13.33333	17	Rainy	exclusion	2.73719
4	CL23	CL23P1	semideciduous	sparse	2.94118	2.94118	17	Rainy	exclusion	2.68485
4	CL23	CL23P2	semideciduous	sparse	9.87654	13.09524	17	Rainy	exclusion	2.68485
4	CL23	CL23P3	semideciduous	sparse	10.00000	9.17431	17	Rainy	exclusion	2.68485
4	CL24	CL24P1	semideciduous	sparse	3.15534	2.91971	17	Rainy	exclusion	2.70243
4	CL24	CL24P2	semideciduous	sparse	10.25641	10.25641	17	Rainy	exclusion	2.70243
4	CL24	CL24P3	semideciduous	sparse	0.00000	4.54545	17	Rainy	exclusion	2.69897
4	CL3	CL3P1	semideciduous	dense	4.61538	4.61538	17	Rainy	open	2.78032
4	CL3	CL3P2	semideciduous	dense	6.25000	9.09091	17	Rainy	open	2.78032
4	CL3	CL3P3	semideciduous	dense	12.00000	18.51852	17	Rainy	open	2.78032
4	CL6	CL6P1	deciduous	sparse	4.76190	23.07692	17	Rainy	open	2.64444
4	CL6	CL6P2	deciduous	sparse	4.00000	25.00000	17	Rainy	open	2.61909
4	CL6	CL6P3	deciduous	sparse	7.14286	31.57895	17	Rainy	open	2.62634
4	CL7	CL7P1	deciduous	sparse	6.25000	58.33333	17	Rainy	open	2.60097
4	CL7	CL7P2	deciduous	sparse	19.60784	33.87097	17	Rainy	open	2.60097
4	CL7	CL7P3	deciduous	sparse	23.52941	38.09524	17	Rainy	open	2.61490
4	CL8	CL8P1	deciduous	dense	0.90909	12.09677	17	Rainy	open	2.67025
4	CL8	CL8P2	deciduous	dense	3.70370	8.45070	17	Rainy	open	2.66087
4	CL8	CL8P3	deciduous	dense	9.75610	13.95349	17	Rainy	open	2.66839
4	CL11	CL11P1	deciduous	dense	7.40741	0.00000	17	Rainy	open	2.76938
4	CL11	CL11P2	deciduous	dense	5.14286	4.04624	17	Rainy	open	2.76492
4	CL11	CL11P3	deciduous	dense	5.88235	27.27273	17	Rainy	open	2.76492
4	CL12	CL12P1	deciduous	semidense	1.44928	0.00000	17	Rainy	open	2.74429
4	CL12	CL12P2	deciduous	semidense	6.89655	0.00000	17	Rainy	open	2.74429
4	CL12	CL12P3	deciduous	semidense	17.30769	10.41667	17	Rainy	open	2.74429
4	CL15	CL15P1	deciduous	semidense	0.00000	16.80672	17	Rainy	open	2.78746
4	CL15	CL15P2	deciduous	semidense	2.70270	28.00000	17	Rainy	open	2.78746
4	CL15	CL15P3	deciduous	semidense	1.69492	21.62162	17	Rainy	open	2.78746
4	CL19	CL19P1	semideciduous	semidense	19.04762	0.00000	17	Rainy	open	3.00260
4	CL19	CL19P2	semideciduous	semidense	8.33333	8.33333	17	Rainy	open	3.00260
4	CL19	CL19P3	semideciduous	semidense	16.66667	6.25000	17	Rainy	open	3.00260

Measure	Cluster	Cod.Plot	Formation	Density	Mort	Recr	Time	Season	Treat	SPrec
4	CL21	CL21P1	semideciduous	dense	7.40741	7.40741	17	Rainy	open	2.73719
4	CL21	CL21P2	semideciduous	dense	11.11111	33.33333	17	Rainy	open	2.73719
4	CL21	CL21P3	semideciduous	dense	20.00000	5.88235	17	Rainy	open	2.73719
4	CL22	CL22P1	semideciduous	dense	16.66667	2.43902	17	Rainy	open	2.73719
4	CL22	CL22P2	semideciduous	dense	11.90476	7.50000	17	Rainy	open	2.73719
4	CL22	CL22P3	semideciduous	dense	3.70370	16.12903	17	Rainy	open	2.73719
4	CL23	CL23P1	semideciduous	sparse	14.28571	3.22581	17	Rainy	open	2.68485
4	CL23	CL23P2	semideciduous	sparse	10.93750	8.80000	17	Rainy	open	2.68485
4	CL23	CL23P3	semideciduous	sparse	12.71186	3.73832	17	Rainy	open	2.68485
4	CL24	CL24P1	semideciduous	sparse	3.50877	19.11765	17	Rainy	open	2.70243
4	CL24	CL24P2	semideciduous	sparse	10.60606	1.66667	17	Rainy	open	2.70243
4	CL24	CL24P3	semideciduous	sparse	7.04225	2.94118	17	Rainy	open	2.69897
5	CL3	CL3P1	semideciduous	dense	9.67742	3.44828	23	Dry	exclusion	1.97772
5	CL3	CL3P2	semideciduous	dense	10.56338	0.00000	23	Dry	exclusion	1.97772
5	CL3	CL3P3	semideciduous	dense	8.53659	0.00000	23	Dry	exclusion	1.97772
5	CL6	CL6P1	deciduous	sparse	45.45455	7.69231	23	Dry	exclusion	1.64345
5	CL6	CL6P2	deciduous	sparse	42.85714	0.00000	23	Dry	exclusion	1.63347
5	CL6	CL6P3	deciduous	sparse	0.00000	0.00000	23	Dry	exclusion	1.64345
5	CL7	CL7P1	deciduous	sparse	31.94444	0.00000	23	Dry	exclusion	1.65321
5	CL7	CL7P2	deciduous	sparse	16.66667	35.48387	23	Dry	exclusion	1.65321
5	CL7	CL7P3	deciduous	sparse	41.50943	6.06061	23	Dry	exclusion	1.66276
5	CL8	CL8P1	deciduous	dense	0.75758	0.75758	23	Dry	exclusion	1.84510
5	CL8	CL8P2	deciduous	dense	18.18182	1.21951	23	Dry	exclusion	1.84510
5	CL8	CL8P3	deciduous	dense	9.56522	0.95238	23	Dry	exclusion	1.86332
5	CL11	CL11P1	deciduous	dense	1.16279	0.00000	23	Dry	exclusion	1.88649
5	CL11	CL11P2	deciduous	dense	9.09091	0.00000	23	Dry	exclusion	1.89209
5	CL11	CL11P3	deciduous	dense	2.24719	0.00000	23	Dry	exclusion	1.89209
5	CL12	CL12P1	deciduous	semidense	23.07692	0.00000	23	Dry	exclusion	2.33846
5	CL12	CL12P2	deciduous	semidense	37.50000	0.00000	23	Dry	exclusion	2.34044
5	CL12	CL12P3	deciduous	semidense	11.88119	0.00000	23	Dry	exclusion	2.33445
5	CL15	CL15P1	deciduous	semidense	3.07692	1.56250	23	Dry	exclusion	1.95904
5	CL15	CL15P2	deciduous	semidense	29.06977	3.17460	23	Dry	exclusion	1.95904
5	CL15	CL15P3	deciduous	semidense	2.32558	2.32558	23	Dry	exclusion	1.95904
5	CL19	CL19P1	semideciduous	semidense	17.39130	0.00000	23	Dry	exclusion	2.36549
5	CL19	CL19P2	semideciduous	semidense	11.11111	0.00000	23	Dry	exclusion	2.36549
5	CL19	CL19P3	semideciduous	semidense	22.85714	0.00000	23	Dry	exclusion	2.36736
5	CL21	CL21P1	semideciduous	dense	4.54545	1.56250	23	Dry	exclusion	1.94939
5	CL21	CL21P2	semideciduous	dense	0.00000	17.85714	23	Dry	exclusion	1.95424
5	CL21	CL21P3	semideciduous	dense	2.17391	16.66667	23	Dry	exclusion	1.94939
5	CL22	CL22P1	semideciduous	dense	7.01754	1.85185	23	Dry	exclusion	1.95424
5	CL22	CL22P2	semideciduous	dense	14.28571	3.22581	23	Dry	exclusion	1.94939
5	CL22	CL22P3	semideciduous	dense	24.44444	0.00000	23	Dry	exclusion	1.94448
5	CL23	CL23P1	semideciduous	sparse	5.88235	0.00000	23	Dry	exclusion	1.87506
5	CL23	CL23P2	semideciduous	sparse	9.52381	0.00000	23	Dry	exclusion	1.87506
5	CL23	CL23P3	semideciduous	sparse	19.26606	1.12360	23	Dry	exclusion	1.87506
5	CL24	CL24P1	semideciduous	sparse	2.18978	0.24814	23	Dry	exclusion	1.89209
5	CL24	CL24P2	semideciduous	sparse	8.97436	4.69799	23	Dry	exclusion	1.89209
5	CL24	CL24P3	semideciduous	sparse	6.06061	3.12500	23	Dry	exclusion	1.89209
5	CL3	CL3P1	semideciduous	dense	8.46154	2.43902	23	Dry	open	1.97772
5	CL3	CL3P2	semideciduous	dense	6.06061	7.46269	23	Dry	open	1.97772
5	CL3	CL3P3	semideciduous	dense	14.81481	0.86207	23	Dry	open	1.97772
5	CL6	CL6P1	deciduous	sparse	23.07692	0.00000	23	Dry	open	1.64345
5	CL6	CL6P2	deciduous	sparse	46.87500	2.85714	23	Dry	open	1.63347
5	CL6	CL6P3	deciduous	sparse	36.84211	7.69231	23	Dry	open	1.64345
5	CL7	CL7P1	deciduous	sparse	11.11111	0.00000	23	Dry	open	1.65321
5	CL7	CL7P2	deciduous	sparse	53.22581	0.00000	23	Dry	open	1.65321
5	CL7	CL7P3	deciduous	sparse	76.19048	61.53846	23	Dry	open	1.66276
5	CL8	CL8P1	deciduous	dense	4.03226	1.65289	23	Dry	open	1.84510
5	CL8	CL8P2	deciduous	dense	8.45070	0.00000	23	Dry	open	1.84510
5	CL8	CL8P3	deciduous	dense	9.30233	0.00000	23	Dry	open	1.86332
5	CL11	CL11P1	deciduous	dense	14.00000	6.52174	23	Dry	open	1.88649
5	CL11	CL11P2	deciduous	dense	2.89017	0.00000	23	Dry	open	1.89209
5	CL11	CL11P3	deciduous	dense	4.54545	0.00000	23	Dry	open	1.89209
5	CL12	CL12P1	deciduous	semidense	1.47059	0.00000	23	Dry	open	2.33846
5	CL12	CL12P2	deciduous	semidense	7.40741	0.00000	23	Dry	open	2.34044
5	CL12	CL12P3	deciduous	semidense	8.33333	2.22222	23	Dry	open	2.33445
5	CL15	CL15P1	deciduous	semidense	4.20168	0.00000	23	Dry	open	1.95904

Measure	Cluster	Cod.Plot	Formation	Density	Mort	Recr	Time	Season	Treat	SPrec
5	CL15	CL15P2	deciduous	semidense	13.00000	0.00000	23	Dry	open	1.95904
5	CL15	CL15P3	deciduous	semidense	14.86486	7.35294	23	Dry	open	1.95904
5	CL19	CL19P1	semideciduous	semidense	5.88235	0.00000	23	Dry	open	2.36549
5	CL19	CL19P2	semideciduous	semidense	16.66667	0.00000	23	Dry	open	2.36549
5	CL19	CL19P3	semideciduous	semidense	9.37500	0.00000	23	Dry	open	2.36736
5	CL21	CL21P1	semideciduous	dense	9.25926	2.00000	23	Dry	open	1.94939
5	CL21	CL21P2	semideciduous	dense	8.33333	0.00000	23	Dry	open	1.95424
5	CL21	CL21P3	semideciduous	dense	11.76471	6.25000	23	Dry	open	1.94939
5	CL22	CL22P1	semideciduous	dense	9.75610	9.75610	23	Dry	open	1.95424
5	CL22	CL22P2	semideciduous	dense	7.50000	0.00000	23	Dry	open	1.94939
5	CL22	CL22P3	semideciduous	dense	3.22581	0.00000	23	Dry	open	1.94448
5	CL23	CL23P1	semideciduous	sparse	16.12903	3.70370	23	Dry	open	1.87506
5	CL23	CL23P2	semideciduous	sparse	7.20000	0.00000	23	Dry	open	1.87506
5	CL23	CL23P3	semideciduous	sparse	11.21495	4.04040	23	Dry	open	1.87506
5	CL24	CL24P1	semideciduous	sparse	10.29412	0.00000	23	Dry	open	1.89209
5	CL24	CL24P2	semideciduous	sparse	16.66667	3.84615	23	Dry	open	1.89209
5	CL24	CL24P3	semideciduous	sparse	7.35294	1.56250	23	Dry	open	1.89209

Supplementary Table S7. Candidate models assessed on mortality and recruitment

ID	Model
1	~1
2	~1 + SPrec
3	~1 + SPrec + Treat
4	~1 + SPrec + Time
5	~1 + SPrec + Treat + Time
6	~1 + SPrec * Treat
7	~1 + SPrec * Treat + Time
8	~1 + SPrec * Time + Treat
9	~1 + SPrec + Treat * Time
10	~1 + Treat
11	~1 + Treat + Time
12	~1 + Treat * Time
13	~1 + Time
